# Estimating seasonal survival of migratory birds using continuous‐time capture–recapture models

**DOI:** 10.1002/eap.70301

**Published:** 2026-09-08

**Authors:** Michael Schaub, Raphaël Arlettaz, Irmi Zwahlen, Ellen C. Martin

**Affiliations:** ^1^ Schweizerische Vogelwarte Sempach Switzerland; ^2^ Conservation Biology, University of Bern Bern Switzerland; ^3^ Conservation Biology, University of Neuchâtel Neuchâtel Switzerland

**Keywords:** carry‐over effect, full annual cycle, migration, mortality hazard rate, seasonal survival, *Upupa epops*

## Abstract

Understanding the population dynamics of migratory species requires consideration of their entire annual cycle. A key requirement is the decomposition of annual survival into seasonal periods. While there is increasing evidence that migrations are the most dangerous phase of the annual cycle, temporal variation in seasonal survival and its effect on population dynamics is still poorly understood. Estimating seasonal survival is challenging when individuals are only encountered during a restricted period of their annual cycle. We developed and tested a continuous‐time capture–recapture model applicable to data from marked individuals that can only be encountered during the breeding season. This model allows annual survival to be separated into breeding and non‐breeding periods. We applied this model to encounter data of hoopoes (*Upupa epops*) collected between 2002 and 2024 from a Swiss population during the breeding period to estimate survival during the breeding and the non‐breeding periods and their respective annual variations. We then linked seasonal survival with environmental variables from the breeding, stopover, and wintering grounds, which have been documented by previous research on the same population. Mortality hazards were 17 times greater during the non‐breeding than during the breeding period, with stronger annual variation observed in the former. Mortality increased during both seasonal periods when a surrogate of prey abundance at the wintering grounds was lower. The effect during the non‐breeding period highlighted that environmental conditions outside the breeding season were an important driver of hoopoe population dynamics, whereas the effect during the breeding period was weaker and provided evidence of a carry‐over effect. In contrast, the environmental variables considered on the breeding and stopover grounds had little impact on seasonal survival. The study adds to the growing body of evidence suggesting that migration and wintering are critical phases in the life cycle of migratory birds and that population dynamics are strongly affected by environmental conditions encountered during wintering. The methodological approach developed is applicable to datasets that record multiple encounters of individuals during the breeding season. It enables the analysis of existing data to study temporal variation in survival across periods of the annual cycle.

## INTRODUCTION

Understanding how animal populations change in abundance and what limits their size are central goals in population ecology and an urgent requirement for conservation. For species living in seasonally variable environments, individuals experience markedly different environmental conditions throughout the year, which can influence demographic rates such as survival and reproduction. Periods of adverse environmental conditions can be lethal to some individuals or reduce their physical condition, potentially affecting survival or subsequent reproductive performance. Although reproduction is energetically demanding for adults and typically occurs when environmental conditions, particularly food availability, are optimal, individuals tend to experience less favorable conditions during the remainder of the year. Because environmental conditions vary throughout the annual cycle, survival may also differ between seasons and can play a key role in shaping population dynamics. To better understand population dynamics, it is necessary to study individuals throughout their annual cycle (Marra et al., 2015; Vickery et al., 2023). This is the only way to assess the effect of seasonal environmental conditions on demographic rates during each period of the annual cycle, as well as of any carry‐over effects from one period to another (Harrison et al., 2011).

In migratory species, seasonal differences are particularly pronounced, as individuals move between regions to exploit favorable conditions, such as tracking seasonal peaks in food supply to reproduce in a given location, while avoiding periods of food scarcity (Alerstam et al., 2003; Somveille et al., 2015). In birds, the migratory period was found to be especially risky for the vast majority of species studied so far, with lower daily survival probabilities than during stationary breeding and non‐breeding periods (Cooper et al., 2024; Klaassen et al., 2014; Newton, 2025; Paxton et al., 2017; Procházka et al., 2017; Rockwell et al., 2017; Rushing et al., 2017; Sergio et al., 2019; Sillett & Holmes, 2002). However, the period with the lowest daily survival probability is not necessarily the period of the annual cycle that has the strongest impact on population dynamics. The contribution of survival at any given period to overall population dynamics depends on the sensitivity of the population growth rate to changes in survival during that period and on the temporal variability of survival (Caswell, 2000). Therefore, to assess the relative impact of the different periods, both temporal variation and mean survival per period must be estimated.

Temporal variation in seasonal survival may originate from variation in environmental conditions. For instance, the probability of black‐tailed godwits (*Limosa limosa*) to successfully cross the Sahara Desert during their spring migration depends heavily on prevailing wind conditions (Loonstra et al., 2019). The amount of rainfall in the wintering grounds was also found to impact either seasonal (Cooper et al., 2024; Rockwell et al., 2017) or annual survival (Millon et al., 2019; Szép, 1995; Woodworth et al., 2017), suggesting a role of rain‐dependent food supply. Due to the difficulty of estimating seasonal survival in migratory populations (see below), most studies are of short duration, with temporal variation rarely the main focus. Consequently, the magnitude of temporal variation in the seasonal survival of migrants and its underlying causes is poorly studied. Therefore, we still know little about how survival during different periods of the annual cycle influences population dynamics (Newton, 2025).

Estimating seasonal survival of migratory populations is challenging because it is difficult to follow mobile individuals throughout the entire annual cycle. When available, high‐resolution data from satellite tracking, which provides information about an individual survival status irrespective of location, may allow seasonal survival to be estimated. These type of data have been instrumental in providing important insights into the ecology of several bird species (Klaassen et al., 2014; Senner et al., 2019; Sergio et al., 2019; Watts et al., 2019). However, satellite transmitters are still relatively inaccessible tools because their large size means they cannot be deployed on the myriad of small species that make up the majority of migratory birds. For smaller birds, ringing thus remains the main classical marking method used to collect capture–recapture data for estimating vital rates (Lebreton et al., 1992). Innovative modeling approaches can still allow the estimation of seasonal survival, but feasibility primarily depends on study design. For instance, if individuals can be encountered twice, one time shortly after arriving at and another time shortly before leaving their breeding grounds, survival during both the breeding period and the rest of the year (the non‐breeding period) can be reasonably estimated (Duriez et al., 2012; Robinson et al., 2020). Yet, it is only when capture–recapture data are collected during the wintering period as well that survival can be jointly estimated during breeding, non‐breeding, and migration (Paxton et al., 2017; Rockwell et al., 2017; Rushing, 2019; Sillett & Holmes, 2002). The sampling design that is applied most often in population studies involves encountering individuals continuously during the breeding period but not outside it. This means that neither of the outlined approaches would be suitable to estimate seasonal survival. We therefore developed a continuous‐time capture–recapture model that is applicable to capture–mark–recapture data collected on the breeding grounds to estimate seasonal, that is, breeding versus non‐breeding, survival in a migratory bird. This approach offers advantages over the classical discrete‐time capture–recapture models. First, there is no need to pool the encounter data into artificial occasions, which can result in the violation of the instantaneous assumption of classical capture–recapture models (O'Brien et al., 2005), and it is more efficient because all encounters can be analyzed. Second, these models are parameterized with hazard mortality rates, allowing for direct comparison of the relative risk of different periods and supporting meaningful covariate modeling and temporal scaling (Ergon et al., 2018).

We first test the developed continuous‐time capture–recapture model to determine its ability to produce unbiased estimates of seasonal mortality and their temporal variability. We then assess how accurately parameters can be estimated when encounter rates vary throughout the breeding season and when arrival and departure dates are known only imprecisely. Secondly, we apply this model to examine the seasonal survival of a Swiss breeding population of hoopoes (*Upupa epops*), which are typical trans‐Saharan migratory birds, over a period of 23 years. Thanks to the deployment of geolocators, the spatiotemporal migration behavior of our hoopoe population could be precisely documented (Liechti et al., 2018; van Wijk et al., 2016, 2017, 2018). This basic information furthermore enables defining seasonal patterns and delineating migratory stopovers and stationary wintering grounds, providing the opportunity to quantify the environmental conditions experienced by the birds. We predict that hoopoes experience lower mortality rates during the breeding period than during the non‐breeding period, particularly given the increased risks associated with spring and autumn migrations. Moreover, we estimate the temporal variability of mortality hazards in the two periods to determine which one affects population dynamics more. Finally, we investigate whether any temporal variation in the occurrence of seasonal hazard rates could be attributed to the environmental conditions experienced during one of the two main periods, and whether there is evidence of carry‐over effects.

## MATERIALS AND METHODS

### The continuous‐time capture–recapture model

We developed a continuous‐time capture–recapture model (Rushing, 2023) to separately estimate survival probabilities during the breeding and the non‐breeding period when detection is impossible during the non‐breeding period. The basic idea is that marked individuals can be encountered multiple times during the breeding period, enabling survival during that period to be estimated. Outside of the breeding period, however, marked individuals cannot be encountered. As surviving individuals may be encountered again in subsequent breeding periods, it is therefore also possible to estimate non‐breeding survival. First, we describe the basic model, and then, we explain how we assessed its performance in estimating parameters.

Capture–recapture models in continuous time are parameterized with the mortality hazard rate (*h*, the instantaneous intensity of deadly events to which an individual is exposed) and the encounter rate or intensity (*p*, the expected number of encounters (or detections) of a given individual in one unit of time, Rushing, 2023). If the mortality hazard rate is constant over a time interval Δ, the survival probability (*s*) over that time interval is calculated as $s=\exp\left(-h\Delta \right)$. For example, if the daily mortality hazard rates is *h* = 0.002, the annual survival probability is exp(−0.002 × 365) = 0.48.

**FIGURE 1:**
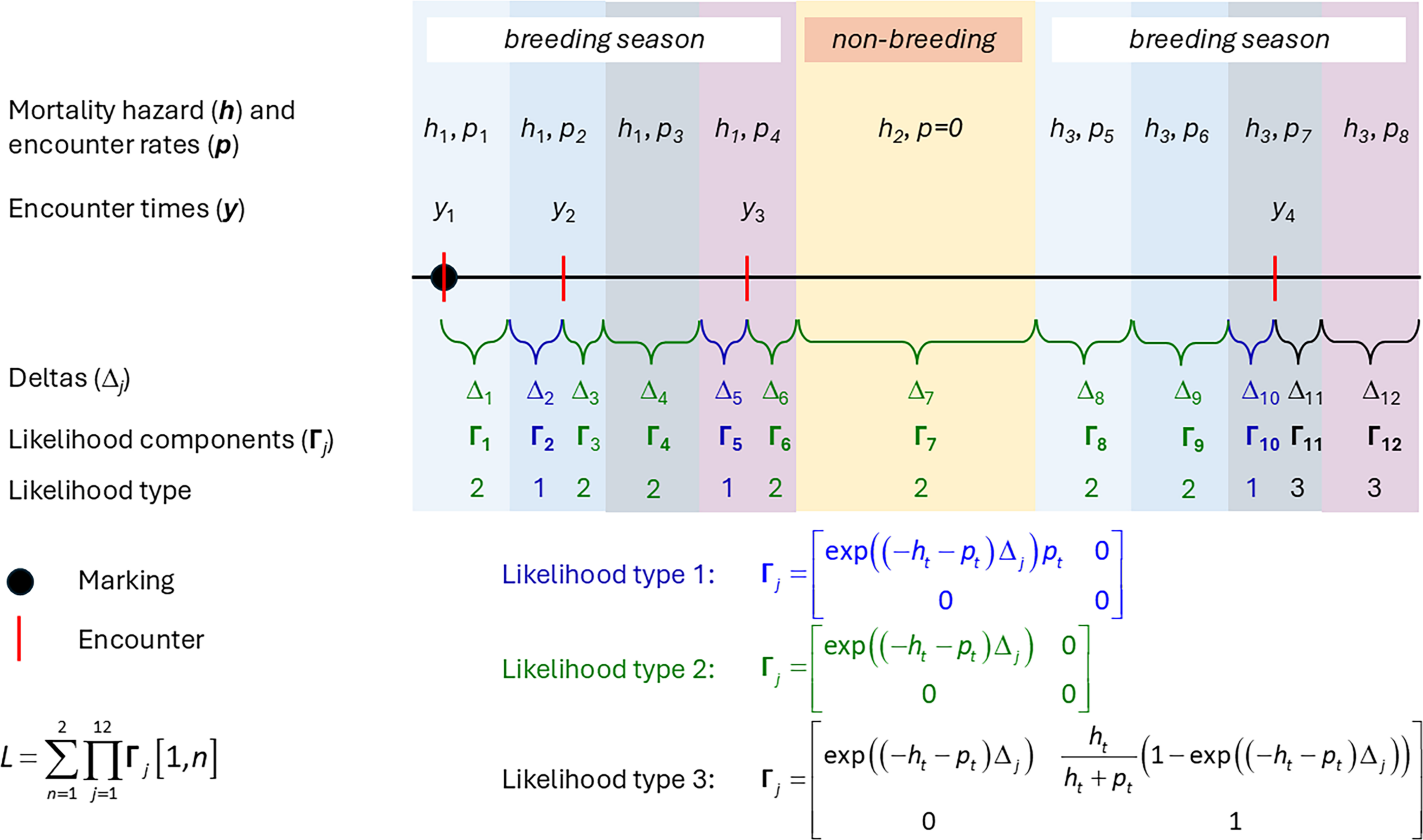
Example of likelihood calculation for an individual that was encountered three times during the breeding season 1 and once during the breeding season 2. The data to be analyzed are the intervals (Δ) calculated from the encounter times and the boundaries of the time intervals (four during the breeding and 1 during the non‐breeding period). For each Δ the likelihood component (Γ) that applies is given, which is a function of mortality hazard (*h*) and encounter rates (*p*) that vary over time (subscripts indicate time). The likelihood of the detection history (*L*) is calculated from the Γ's.

The basic data used are lists of encounter dates for each individual (detection histories). To estimate time‐varying hazard and encounter rates, dates at which rates are assumed to change must be defined, forming time intervals. Within each time interval, both the mortality hazard and the encounter rates are constant. From the encounter dates, the differences (1) between consecutive capture dates within a given time interval, (2) between the last encounter date within a time interval and the end of that time interval, and (3) between the start of a time interval and the first encounter within that time interval are calculated (Δ, Figure 1). These data are then used to estimate mortality hazard and encounter rates for each time interval.

We adopt a multistate model with the states “alive” and “dead” (Rushing, 2023) and define the transition matrix between these states as $Q=\left[\begin{array}{cc} -h & h\\ 0 & 0 \end{array}\right]$. The probability of transition after some time $\Delta =t_{2}-t_{1}$ is calculated as $\exp\left\{Q\Delta \right\}$, where exp{} denotes the matrix exponential, and requires *h* to be constant during the interval Δ. We then define the detection vector $P=\left[p\;0\right]$ whose elements are the encounter rates of the two states “alive” and “dead.” The state‐specific probability to encounter a marked individual after time Δ (i.e., right at *t*_2_, but not between *t*_1_ and *t*_2_) is $\exp\left\{-D\left(P\right)\Delta \right\}D\left(P\right)$, where *D*(***P***) is the diagonal matrix whose diagonal is ***P***. The encounter rate *p* needs to be constant during Δ. The probability to survive and be encountered after some time Δ is then computed as $\Gamma =\exp\left\{\left(Q-D\left(P\right)\right)\Delta \right\}D\left(P\right)$, and the probability to not encounter an individual in an interval Δ is $\Gamma =\exp\left\{\left(Q-D\left(P\right)\right)\Delta \right\}$. Based on these reasonings, we can compute the different likelihood components that make up an individual's encounter history. Because *h* and *p* need to be constant during any Δ, we need to specify time boundaries defining periods with constant rates and calculate the Δ from the encounter dates relative to these boundaries. There are three types of likelihood components, depending on the individual's detection status at the end of a time interval: (1) the individual was detected at the end of the interval; (2) the individual was not detected at the end of the interval but was known to be alive because it was encountered again later; and (3) the individual was not detected at the end of the interval, and its survival status remained unknown. Because the matrices whose exponential needs to be calculated are diagonalizable, expressions could be derived for these three likelihood components (Figure 1). The likelihood of a detection history is computed as a function of all the components, as shown in Figure 1. The variant of the model in focus here assumes that detections are impossible during certain time intervals, consequently fixing the corresponding encounter rates to zero. Specifically, we assume that encounters are possible during a breeding period, during which the encounter rate may or may not be temporally variable. Then, during the non‐breeding period, encounters are impossible and the encounter rate is fixed to zero. The aim is to estimate the mortality hazard rates during each period, that is, seasonal survival.

### Simulation study to assess the performance of the model

If encounters had been possible during the breeding and non‐breeding periods, there is no doubt that the model would be able to estimate the hazard and encounter rates during these periods (Rushing, 2023). However, as we had no encounters during the non‐breeding period, it was not a priori clear whether the two seasonal hazard rates could be estimated independently, and whether the temporal variation in these rates could be accurately recovered. We therefore conducted a simulation study to investigate three aspects of the model used. First, we examined whether the parameters can be estimated when the data generation model is used to analyze the data. The main question here is whether the mortality hazard during the non‐breeding period, when there are no observations, can be estimated accurately. Furthermore, we transformed the simulated data to binary, discrete‐time capture histories and applied a Cormack‐Jolly‐Seber (CJS) model (Lebreton et al., 1992) to estimate annual survival. We then compared these estimates with annual survival derived from the seasonal mortality hazard rates. Second, we examined the extent to which the mortality hazard and encounter rates are biased when the temporal pattern of encounter rates during the breeding period is incorrectly specified. Third, we evaluated potential bias in the target parameters when the assumptions that arrival and departure dates used to define the time intervals are identical across all individuals and known without error are violated. The first is therefore a question of general parameter accuracy and identifiability. The second and third are about the consequences of violating some model assumptions. All are evaluated by simulations.

#### General parameter accuracy and identifiability

The simulations are inspired by the current hoopoe study. We defined the breeding period as 132 days and the non‐breeding period as 233 days. For each simulation, mean hazard rates for the breeding and non‐breeding periods were generated from uniform distributions ranging from 0.0015 to 0.003, and temporal SDs were generated from uniform distributions ranging from 0.1 to 0.5. We then used normal distributions with the generated means and SDs to create annually varying breeding and non‐breeding hazard rates. Similarly, we created encounter rates for the breeding period, with a mean from a uniform distribution ranging from 0.005 to 0.01 and SDs from a uniform distribution ranging from 0.1 to 0.5. The annually varying encounter rates were then generated from the created mean and SD. Based on these values, and assuming that 100 individuals were marked each year for 10 years, we simulated 200 datasets. Each dataset was analyzed using the data‐generating model that employed temporal (i.e., among years) random effects for the breeding and non‐breeding hazard rates, as well as for the breeding encounter rates. We compared the posterior means of the parameters with the parameters that generated the data to evaluate the estimation performance of the model.

#### Model performance under violating assumptions

We were first interested in investigating the effect on parameter estimates if the timing and duration of the time intervals for the encounter rates (thereafter temporal pattern) during the breeding period were incorrectly specified in the model, that is, if the data generation model did not match the data analysis model. We used two scenarios. First, we assumed that the encounter rate was higher in the first half of the breeding period than in the second. Secondly, we assumed the reverse pattern, that the encounter rate was lower in the first period than in the second. In both scenarios, we assumed that the mortality hazard differed between the breeding and non‐breeding periods, but that there was no annual variation and the data generation values were also the same for each simulation. We simulated 200 datasets for each scenario that were analyzed each with different models as described below.

We analyzed the resulting datasets using four models. In all models, the hazard rates were estimated using two parameters that remained constant over time. Four models were used for the encounter rates, which differed in their temporal pattern during the breeding period. Firstly, we assumed a single value, that is, a constant encounter rate throughout the breeding period. Secondly, we assumed that the breeding period was divided in half, with each half having its own encounter rate. Thirdly, the breeding period was divided into three equal‐length time intervals, with different encounter rates estimated for each. Finally, the breeding period was divided into four equal‐length time intervals, with different encounter rates estimated for each. Models 1 and 3 violated the model assumptions; Model 1 did so to a greater extent than Model 3. Models 2 and 4 did not violate the model assumptions; however, Model 4 was less parsimonious than Model 2, which was the data‐generating model.

Secondly, we examined how violating the assumption of uniform and known arrival and departure dates among individuals affects outcomes. Simulations included moderate and strong individual variation in arrival and departure dates, as well as scenarios with specified breeding periods that were too short or too long compared to reality (see Appendix S1: Section S1 for details). A baseline scenario meeting all assumptions was also considered. Each scenario involved simulating and analyzing 200 datasets.

We used the Bayesian framework to analyze the simulated data and used software NIMBLE (de Valpine et al., 2017) for model fitting. We defined distributions that made use of our custom‐defined likelihood for the continuous‐time capture–recapture model and used these distributions in NIMBLE. We defined vague priors for all parameters. To analyze the data generated we ran two Monte Carlo Markov chains (MCMC) in all simulation studies, but with slightly different specifications: we generated 6000 samples, discarded the first 3000 as a burn‐in and kept every third sample for the first simulation. For the second and third simulations, we generated 4000 samples of which we discarded the first 1000 and kept every second of the remaining ones. These specifications yielded 2000 posterior samples from converged MCMC chains as based on R‐hat statistics (Brooks & Gelman, 1998). Code for these analyses is available on the vogelwarte.ch Open Repository and Archive https://doi.org/10.5281/zenodo.20021072 (Schaub et al., 2026).

### The hoopoe case study

#### Study species and study area

**FIGURE 2:**
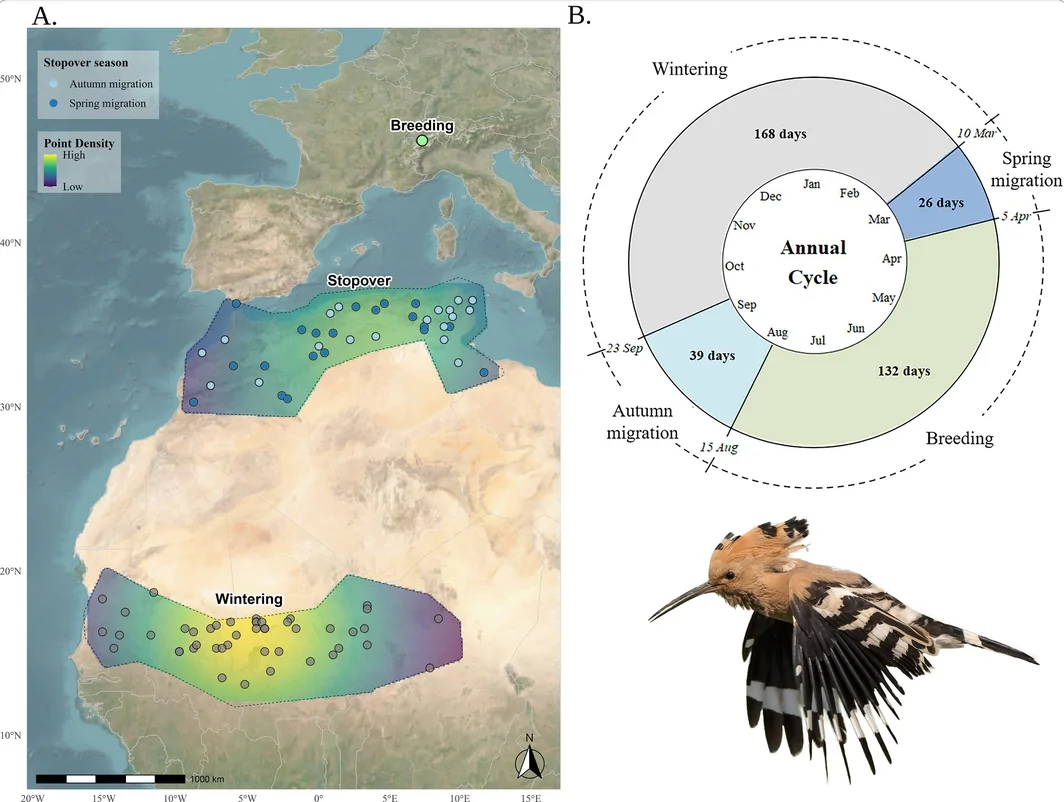
(A) Location of the hoopoe breeding population, its stopover grounds during autumn and spring migration, and its wintering grounds. Dots indicate stationary locations of adult hoopoes tracked with geolocators (data source: Schaub et al., 2025). The polygons indicate the areas from which the Normalized Difference Vegetation Index (NDVI) values were obtained. (B) Annual cycle of the hoopoes, as used in the analysis. The dates demarcating the different periods are the mean dates extracted from the geolocator‐tracked hoopoes (van Wijk et al., 2017). Photo credit: Marcel Burkhardt.

The hoopoe is a small (mean body mass about 75 g) migratory, insectivorous bird species that inhabits semi‐open farmland that we studied on the plain of the Rhône valley in southwestern Switzerland (canton of Valais, 46°14′ N, 7°22′  E, 450–520 m above sea level) from 2002 to 2024. The 62‐km^2^ study area is intensively cultivated with fruit tree plantations and vineyards and is characterized by warm and dry summers. Hoopoes breed in cavities and readily accept nest boxes when natural cavities are lacking. Starting in 1999, we installed approximately 700 nest boxes in the study area, which initially led to a sharp increase in the population size, followed by a stabilization (Arlettaz, Schaub, Fournier, et al., 2010; Plard et al., 2020). Hoopoes prey mainly on ground‐dwelling invertebrates such as molecrickets (*Gryllotalpa gryllotalpa*; Arlettaz, Schaub, Reichlin, et al., 2010). Typical brood sizes range from four to six nestlings and about one third of all females raise a second brood (Hoffmann et al., 2015). The hoopoe is short‐lived with an annual adult survival probability of about 0.4 (Schaub et al., 2012). Thanks to previous studies using geolocators, the migration behavior of the hoopoes breeding in our study area is well documented (Liechti et al., 2018; Schaub et al., 2025; van Wijk et al., 2016, 2017, 2018). Hoopoes spend the stationary wintering portion of the non‐breeding period in the Sahel south of the Sahara Desert in Africa, at locations that extend over a very large area ranging from Senegal to Nigeria (Figure 2). They reach these areas via different flight routes of about 3700 km. Adults leave the breeding grounds on average on 15 August and fly along different routes to the wintering grounds, where they arrive, on average, on 23 September (van Wijk et al., 2017). Hoopoes remain largely stationary in their wintering grounds and start spring migration on 10 March, on average, resulting in a total duration of 168 days spent on the wintering grounds. The mean arrival date on the breeding grounds is 5 April, so the spring migration journey is on average faster (26 days) than the fall migration journey (39 days). The duration of the two migrations plus the stationary wintering period (i.e., non‐breeding period) sums up to 233 days, while the average breeding period lasts for 132 days (36% of the year).

#### Data collection

Data were collected from 2002 to 2024. All nest boxes were checked every second week during the breeding season, and nest boxes occupied by hoopoes were inspected approximately every fifth day to collect information on breeding parameters. Hoopoe nestlings were ringed when they were about 12 days old, and adults involved in a breeding attempt were captured once with mist nets or spring traps after the chicks had hatched. From 2002 to 2016, we used standard bird rings. From 2017 onward, all treated hoopoes received, in addition to the standard ring, an alphanumeric color ring. To monitor breeding adults, we installed motion‐triggered cameras on occupied nest boxes for 1 day starting in 2018. This was done immediately after the detection of an active brood, not only after hatching of the chicks as was previously the case. Unmarked adult individuals were physically captured and marked. Since this capture–mark–recapture data were collected only from the breeding grounds, mortality and permanent emigration from the breeding grounds cannot be separated, resulting in estimates of apparent survival (Lebreton et al., 1992). While a significant proportion of locally born hoopoes emigrate to other populations (Schaub et al., 2012), breeding dispersal is weak (Bötsch et al., 2012). As only a small proportion of locally breeding adults permanently leave the study area, apparent survival is likely to be close to true survival, and we therefore focused on adult survival only. Locally born juveniles were considered in our analysis only if they were later encountered as adults in the study area, and only from that time onward. In total, 2051 individuals were encountered between one and 16 times in total, and up to five times per individual within a breeding season.

#### Environmental variables

We quantified environmental conditions for locations that represented four biologically relevant phases of the annual cycle of the hoopoes: the breeding grounds in Switzerland, the wintering grounds, and the stopover grounds on autumn and spring migration. Environmental variables from the breeding grounds included temperature and precipitation (Appendix S1: Section S2). Both covariates have been directly linked to key hoopoe demographic processes during reproduction, with positive effects of temperature detected on juvenile survival and reproductive success (Arlettaz, Schaub, Reichlin, et al., 2010; Plard et al., 2020). We used the first principal component (PC) from a PC analysis using the two variables, which was positively correlated with temperature and negatively with precipitation. The environmental variable considered on the wintering grounds and the migratory stopover grounds was the Normalized Difference Vegetation Index (NDVI). NDVI is a measure of greenness and is a valuable proxy for vegetation cover, that is, mirrors to a large extent ecosystem quality and resource availability (Pettorelli et al., 2005). NDVI is particularly informative in arid regions, where vegetation dynamics respond strongly to rainfall, which likely drives the abundance of arthropods. It is generally considered as a reliable indicator for arthropod abundance in the Sahel (Zwarts et al., 2009). For example, locust (*Acrididae* sp.) density, a potential prey of hoopoes, is positively correlated with NDVI (Schlaich et al., 2016). We derived standardized annual values of NDVI for these locations (wintering: NDVI^w^; autumn migration: NDVI^a^; spring migration: NDVI^s^). Details on the derivation are given in Appendix S1: Section S2.

#### Data analysis

We used the developed continuous‐time capture–recapture model to analyze the hoopoe data. To estimate seasonal hazard rates and temporally variable encounter rates during the breeding period, we defined specific dates at which rates were assumed to change. The breeding and non‐breeding periods were defined by the mean dates of arrival on and departure from the breeding grounds (5 April and 15 August) and last 132 and 233 days, respectively. As the encounter rate was likely to change during the course of breeding—due to different reproductive activities, asynchronous timing between broods and therefore variation in our ability to encounter individuals—we assessed several encounter rate variants (Appendix S1: Section S3). The most supported variant, and the one used in our model, considered four different encounter rates throughout the breeding period, with some interannual variation. In the first time interval of 22 days (5–27 April), early breeding individuals could be encountered. In the second time interval of 22 days (27 April–19 May), most breeding pairs had started their brood. The third time interval of 55 days (19 May–13 July) corresponded to high breeding activity, when most individuals were involved in a brood and when most second broods had started. During the last time interval of 33 days (13 July–15 August), late breeders could still be encountered. We assumed that the mortality hazard rate remained constant during these four time intervals of the breeding period but was different to that during the non‐breeding period. From the hoopoe encounter dates, the differences (1) between consecutive capture dates within a given time interval, (2) between the last encounter date within a time interval and the end of that time interval, and (3) between the start of a time interval and the first encounter within that time interval were calculated (Δ, Figure 1). These data were then used to estimate the parameters.

As for the simulation study, we used software NIMBLE to analyze the hoopoe data. We defined vague priors for all parameters (Appendix S1: Section S4). We ran three MCMC for 25,000 iterations, discarding the first 5000 as the burn‐in period and retaining every second value. This specification resulted in an MCMC sample size of 30,000. We checked the convergence of the MCMC chains visually and by using *R*‐hat statistics (Brooks & Gelman, 1998). To characterize the resulting posterior distributions, we generally report posterior medians and the 95% highest density intervals (HDIs). Data and code for the analyses are available on the vogelwarte.ch Open Repository and Archive https://doi.org/10.5281/zenodo.20021072 (Schaub et al., 2026).

#### Hypotheses and modeling strategy

We fitted different models for different purposes. First, we fitted a model without covariates (denoted as *h* ^*B*^(*t*), *h* ^*N*^(*t*)), in which the breeding and non‐breeding period mortality hazard rates were permitted to vary independently of each other across years, with the years treated as random effects. Therefore, the mortality hazard rate *h*_*t*,*s*_ in year *t* and season *s* was modeled as $\log\left(h_{t,s}\right)\sim \text{Normal}\left(\log\left({\bar{h}}_{s}\right),\sigma_{s}^{2}\right)$, where ${\bar{h}}_{s}$ and $\sigma_{s}^{2}$ were the season‐specific means and annual variations of the mortality hazard rates, respectively. Encounter rates during the breeding period were modeled with an interaction between the time intervals and the year, with the latter treated as a fixed effect. This first model was used to assess which seasonal period was more dangerous for hoopoes, as well as to determine the temporal patterns of mortality. We computed seasonal survival probabilities from the seasonal mortality hazard rates as $s_{t,s}=\exp\left(-h_{t,s}\Delta_{s}\right)$, where $\Delta_{s}$ was the duration of each period (132 and 233 days, respectively). We also calculated the annual survival probability as the product of the two seasonal survival probabilities.

The second step aimed to establish whether environmental variables could explain the annual variation in seasonal mortality hazard rates. To this end, we defined linear models for the hazard rates as $\log\left(h_{t,s}\right)\sim \text{Normal}\left(\log\left({\bar{h}}_{s}\right)+\beta x_{t},\sigma_{s}^{2}\right)$, where β was a parameter to be estimated and *x*_*t*_ was a standardized environmental variable.

We formulated the following hypotheses regarding the impact of the four environmental covariates on seasonal mortality hazard rates. The vegetation index from the wintering grounds (NDVI^w^) could negatively impact both the non‐breeding and breeding hazard rates. The former was a direct consequence of locally prevailing environmental conditions, while the latter was a carry‐over effect. It was expected that the vegetation index on the autumn migration stopover grounds (NDVI^a^) would have a negative impact on the hazard of non‐breeding mortality, but it was unlikely to operate as a carry‐over effect on breeding mortality more than a half year later. Similarly, the vegetation index on the spring migration stopover grounds (NDVI^s^) may have affected breeding mortality, but it was unlikely to impact the following non‐breeding mortality. Finally, wet and cold weather during the breeding period has a negative effect on the reproductive success of hoopoes (Arlettaz, Schaub, Reichlin, et al., 2010). We therefore hypothesized that weather conditions during the breeding period, as reflected by the first PC of mean temperature and precipitation, might affect both breeding and non‐breeding mortality. The latter represented a carry‐over effect and may occur because hoopoes may have depleted their energy reserves after raising broods in adverse weather conditions, thereby increasing mortality during autumn migration. We formulated seven models, each representing one of the hypotheses. No model was formulated for relationships that were considered unlikely.

To make inferences, we performed model selection in two steps using the widely applicable Akaike information criterion (WAIC) and the probability of a negative slope parameter (*P*). First, we compared six univariate models, each of which included only one environmental covariate and reflected one of the above‐described hypotheses. We also included the model without covariates. We then selected the covariates of the best fitting models (defined as ΔWAIC < 2 and *P* > 0.8) and used them to construct further models incorporating multiple covariates (i.e., all possible combinations of the selected covariates). In this second step, we selected the best overall model for inference.

## RESULTS

### Assessment of the continuous‐time capture–recapture model

#### Performance of the model

**FIGURE 3:**
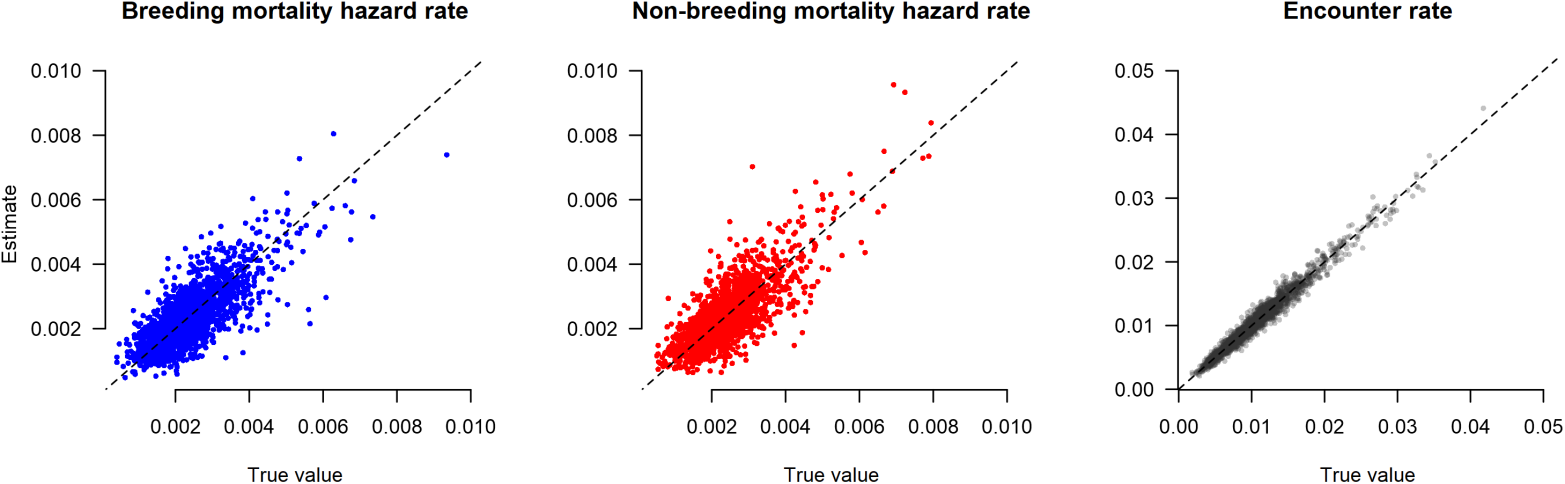
Posterior means of the mortality hazard rates and encounter rates against the data‐generating values under a model with random time effects for hazard and encounter rates. Each point in each panel refers to an annual value (10 for breeding season hazard and encounter rates, 9 for non‐breeding season hazard rates) of each of the 200 simulations.

The continuous‐time capture–recapture model produced estimates of seasonal breeding and non‐breeding hazard rates, as well as encounter intensities, that aligned very well with the parameters used to generate the data (Figure 3). The relative biases of the annual, seasonal‐specific mortality hazards, as well as their long‐term means, were virtually zero (Appendix S1: Figure S1). This indicates that the model produced reliable estimates that were identifiable when the model assumptions are met. We also computed the annual survival probabilities from the seasonal mortality hazard rates and found these to be unbiased, too. In contrast, annual survival estimates obtained from a CJS model (Lebreton et al., 1992) based on annual binary data exhibited a slight negative bias (Appendix S1: Figure S2). The annual survival probabilities obtained from the continuous‐time capture–recapture model were also more precise than those obtained from the CJS model.

#### Temporal variation in encounter intensities

**FIGURE 4:**
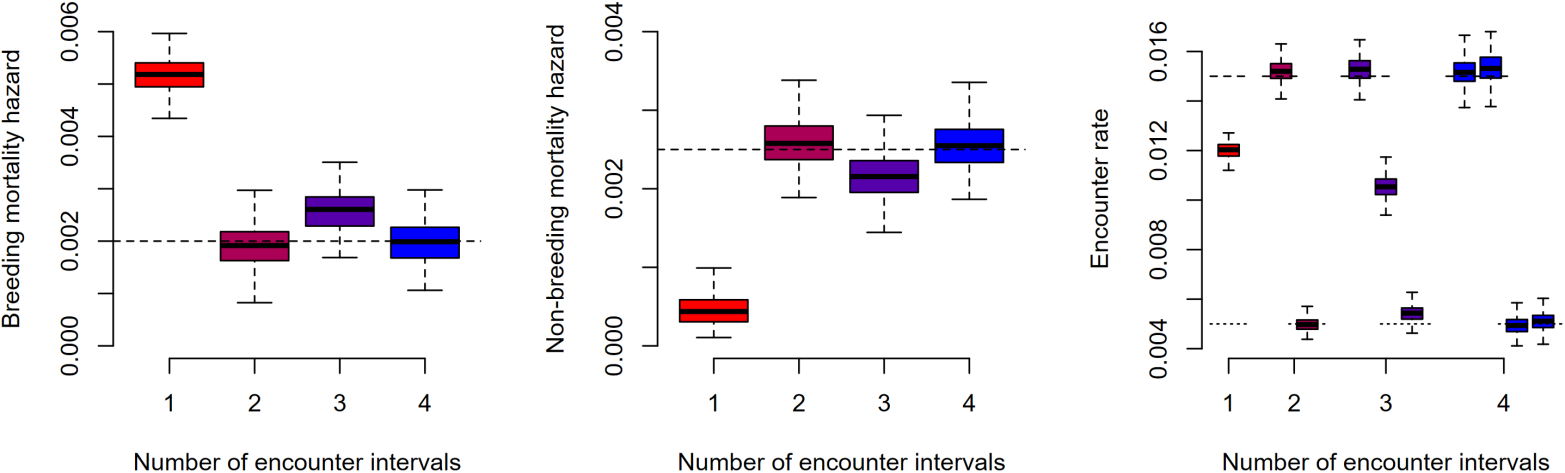
Box plots of posterior means (across simulations) of seasonal mortality hazards and encounter rates obtained from models considering different numbers of encounter intervals during breeding (four models represented by four colors). The broken lines show the true values. The graph shows results from Scenario 1, where the true encounter rate is higher in the first than in the second interval. Results under Scenario 2 are shown in Appendix S1: Section S1.

The simulation showed that estimates of mortality hazards were biased if the temporal pattern of encounter intensities was not accurately reflected (Figure 4, Models 1 and 3). The largest bias occurred in Model 1, which assumed that encounter rates remained constant throughout the breeding period, when in fact they differed between the first and the second half of the breeding period. The direction of the bias depended on the scenario. If in reality the encounter rate was higher in the first interval of the breeding period than in the second, the breeding mortality hazard was positively biased and the non‐breeding mortality hazard was negatively biased (Figure 4). The reverse pattern was found when the encounter rate was actually lower in the first interval than in the second (Appendix S1: Figure S3). When three time intervals of encounter rates were specified in the model, the pattern of bias in the mortality hazards remained the same, but the bias was much reduced (Figure 4). The mortality hazard estimates were unbiased in both scenarios if either the correct number of encounter rates was specified (Model 2) or if a larger number of encounter rates were used that captured the correct temporal pattern (Model 4, Figure 4). SDs of mortality hazards increased with an increasing number of intervals during the breeding period (Appendix S1: Figure S4). The main difference occurred when only one interval was considered versus at least two, but there was not much change when more intervals were considered. Interestingly, mortality hazard rates were estimated more accurately when the encounter rate was higher in the second breeding period interval. It seems that there was an advantage when detection was high just before the start of the non‐breeding period, during which time no detections occurred.

The simulation showed that adequately capturing the temporal pattern of encounter rates during the breeding period is important for the accurate estimation of seasonal mortality hazards. Using a model that allows temporal flexibility in encounter rates will reduce the bias, even if the temporal pattern is not entirely correct. Therefore, it is recommended that enough temporal resolution is used when specifying models for encounter rates. While the price in terms of reduced precision appears to be minimal (Appendix S1: Figure S4), computation time increases significantly with the number of intervals considered.

#### Variable and uncertain arrival and departure dates

**FIGURE 5:**
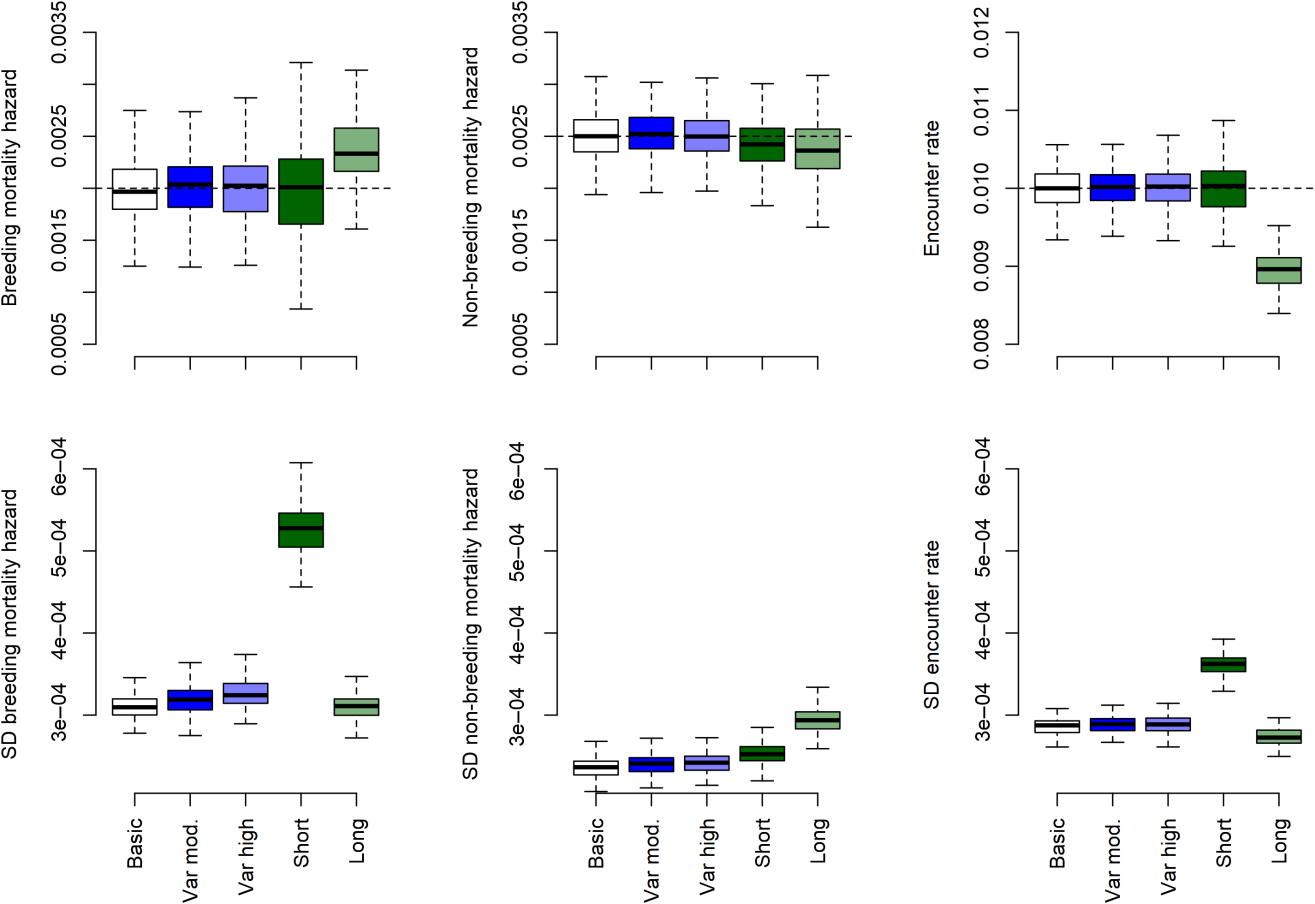
Boxplots of posterior means (across simulations, top row) and SDs (bottom row) of seasonal mortality hazards and encounter rates obtained under five scenarios about arrival and departure dates. Basic (constant and known perfectly), Var. mod (moderate individual variability in arrival/departure dates), Var. strong (strong individual variability in arrival/departure dates), Short (breeding period assumed to be too short), and Long (breeding period assumed to be too long). The broken lines show the true values.

When arrival and departure dates and therefore the length of breeding and non‐breeding periods varied among individuals, estimates for mortality hazard and encounter rates were nearly unbiased, even though the model assumed identical period lengths for all individuals (Figure 5). Parameter uncertainty increased slightly as variability in arrival and departure dates grew. If the breeding period was incorrectly considered shorter than it actually was, estimates for mortality hazard and encounter rates during breeding remained unbiased, but estimation uncertainty increased (Figure 5). The mortality hazard rate during the non‐breeding period exhibited a small negative bias. If the breeding period was assumed to be longer than reality, both mortality hazard and encounter rates during breeding became positively and negatively biased, respectively. Mortality hazard rates during non‐breeding were also negatively biased, though to a lesser extent. The resulting annual survival probabilities were nearly unbiased in all scenarios (Appendix S1: Figure S5).

### Hoopoe case study

#### Mortality in the different periods and temporal patterns

The mean daily mortality hazard rate during the non‐breeding period, estimated from the model without environmental covariates (*h* ^*B*^(*t*), *h* ^*N*^(*t*)), was 36.123 × 10^−4^ (HDI: 31.273 × 10^−4^–40.051 × 10^−4^), which was 17.3 (HDI: 1.0–293.1) times higher than during the breeding period (2.098 × 10^−4^; HDI: 0.000–8.882 × 10^−4^). These mortality hazard rates translated into survival probabilities of 0.43 (HDI: 0.39–0.48) and 0.97 (HDI: 0.89–1.00) during the non‐breeding and the breeding periods, respectively, resulting in an annual mean adult survival probability of 0.42 (HDI: 0.39–0.45). Only 3.2% (HDI: 0.0–13.3) of lethal events occurred during the breeding period, compared to 96.8% (HDI: 86.7–100.0) during the non‐breeding period (Table 1). Therefore, the non‐breeding period, which included the two migration routes, was significantly riskier than the breeding period.

**TABLE 1:** Period‐specific mean hazard rates from the basic model (*h* ^*B*^(t), *h* ^*N*^(t)), resulting survival probabilities (over the complete period) and proportion of experienced mortality.

Season	Hazard rate (×10^−4^)	Survival (complete period)	Proportion of mortality	Duration (days)
Breeding season	2.10 (0.00–8.88)	0.97 (0.89–1.00)	0.03 (0.00–0.13)	132
Non‐breeding season (total)	36.12 (3.13–40.05)	0.43 (0.39–0.48)	0.97 (0.87–1.00)	233
Autumn migration	102.39 (91.36–144.51)	0.62 (0.57–0.70)	0.56 (0.42–0.60)	39
Wintering (stationary)	2.10 (0.00–8.88)	0.97 (0.86–1.00)	0.04 (0.00–0.17)	168
Spring migration	102.39 (91.36–144.51)	0.72 (0.69–0.79)	0.37 (0.28–0.40)	26

*Note*: The splitting of the non‐breeding period into periods of spring and autumn migration and wintering is based on the assumptions that the mortality hazard during breeding is the same as during the stationary wintering period and that the hazard rates on both migrations are the same. Given are posterior medians and the 95% highest density credible intervals (in parentheses). Note that for better readability the values given for the hazard rates must be multiplied by 10^−4^ to get the real values.

**FIGURE 6:**
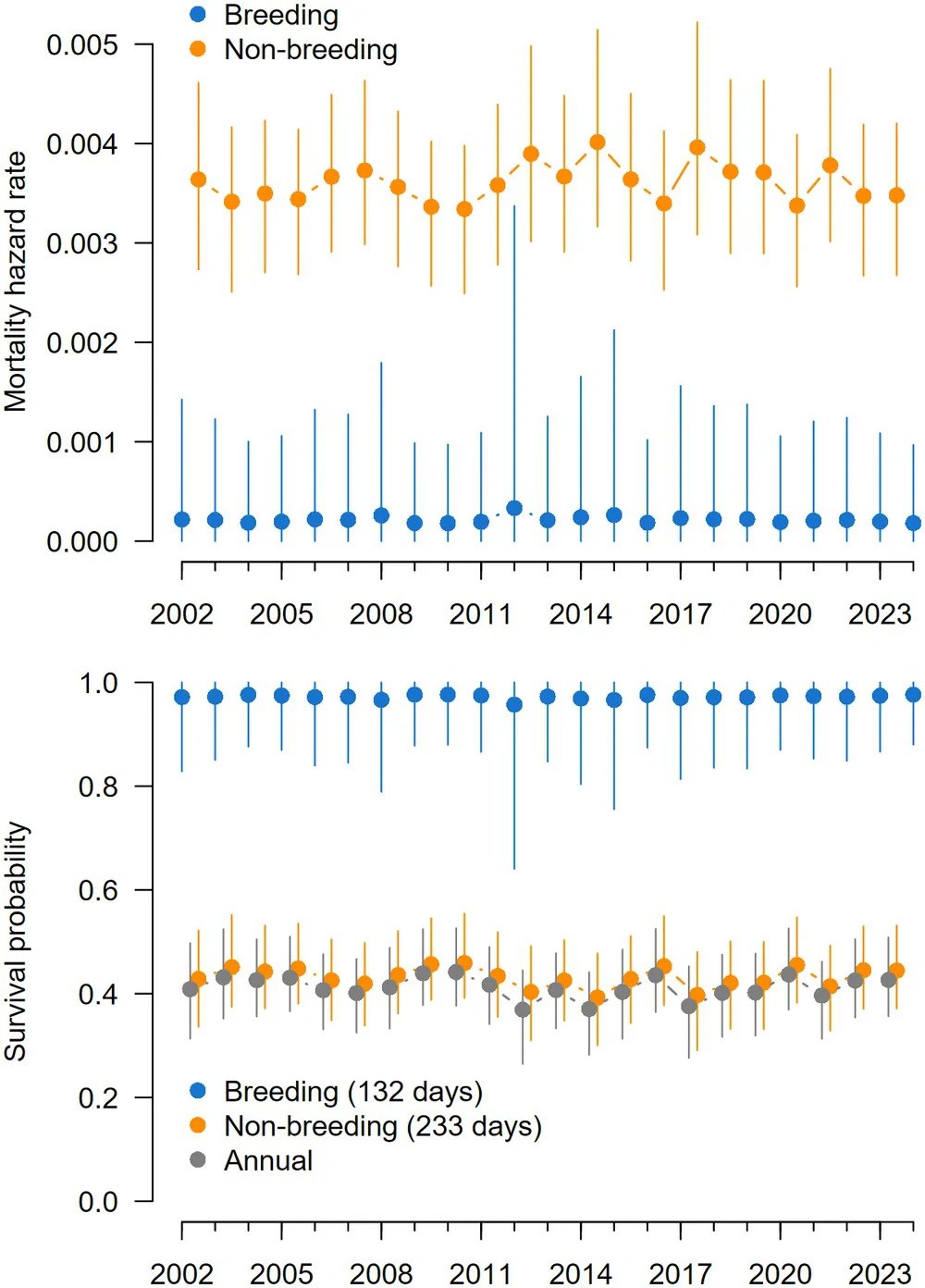
Estimated daily mortality hazard rates (upper panel) and derived survival probabilities (lower panel) of adult hoopoes during the breeding and the non‐breeding season based on the model without covariates (*h* ^*B*^(*t*), *h* ^*N*^(*t*)). The annual survival probabilities are also shown. The dots are posterior medians and the vertical lines show the 95% highest density intervals.

The annual mortality hazard rates during the breeding and non‐breeding periods varied between years without showing a clear trend (Figure 6). The estimated temporal variance in log hazard rates was greater during the breeding period (0.93; HDI: 0.02–2.41) than the non‐breeding period (0.12; HDI: 0.01–0.22; Appendix S1: Section S5). However, the resulting period‐specific survival varied more during the non‐breeding period than the breeding period, mirroring the temporal pattern of annual survival very closely (Figure 6).

#### Impact of environmental variables on mortality hazards

In the first modeling step, we compared single‐variable models (Table 2). The best model included the effect of winter NDVI on the non‐breeding hazard rate. Based on our criteria (ΔWAIC < 2 and *P* > 0.8), we also retained the effect of winter NDVI on the breeding mortality hazard rate and the effect of breeding weather (PC) on the non‐breeding mortality hazard rate.

**TABLE 2:** Model selection results for the impact of environmental covariates (NDVI^w^: scaled average annual NDVI on the wintering grounds; NDVI^s^: scaled average NDVI on spring migration stopover grounds; NDVI^a^: scaled average NDVI on autumn migration stopover grounds; PC: first principal component of breeding temperature and amount of rainfall) on mortality hazards during the breeding period (*h* ^*B*^) and the non‐breeding period (*h* ^*N*^).

Model	lppd	pWAIC	ΔWAIC	β	*P*
*h* ^*B*^(t), *h* ^*N*^(t)	−12826.56	70.95	1.04	…	…
*h* ^*B*^(t), *h* ^*N*^(NDVI^w^)	−12825.31	71.68	0.00	−0.067 (−0.160; 0.015)	0.94
*h* ^*B*^(NDVI^w^), *h* ^*N*^(t)	−12826.31	70.87	0.37	−0.511 (−1.637; 0.672)	0.84
*h* ^*B*^(NDVI^s^), *h* ^*N*^(t)	−12825.93	71.17	0.22	−0.255 (−1.441; 0.831)	0.69
*h* ^*B*^(t), *h* ^*N*^(NDVI^a^)	−12826.03	71.88	1.84	−0.002 (−0.084; 0.081)	0.52
*h* ^*B*^(PC), *h* ^*N*^(t)	−12826.29	70.93	0.45	0.065 (−1.285; 1.298)	0.46
*h* ^*B*^(t), *h* ^*N*^(PC)	−12825.95	71.51	0.93	−0.048 (−0.139; 0.047)	0.85

*Note*: The following is provided for each model: model name; log predictive density (lppd); estimated effective number of parameters (pWAIC); difference in the widely applicable Akaike information criterion between the current and the best model (ΔWAIC); estimated regression coefficient of the environmental covariate on the seasonal mortality hazard rate (posterior median and 95% highest density interval, β); and probability that the effect is negative (*P*). Also shown is the basic model without covariates (*h* ^*B*^(t), *h* ^*N*^(t)). All models included annual random effects for the two hazard rates, and the model for the encounter rate was always the same as described in the text.

**FIGURE 7:**
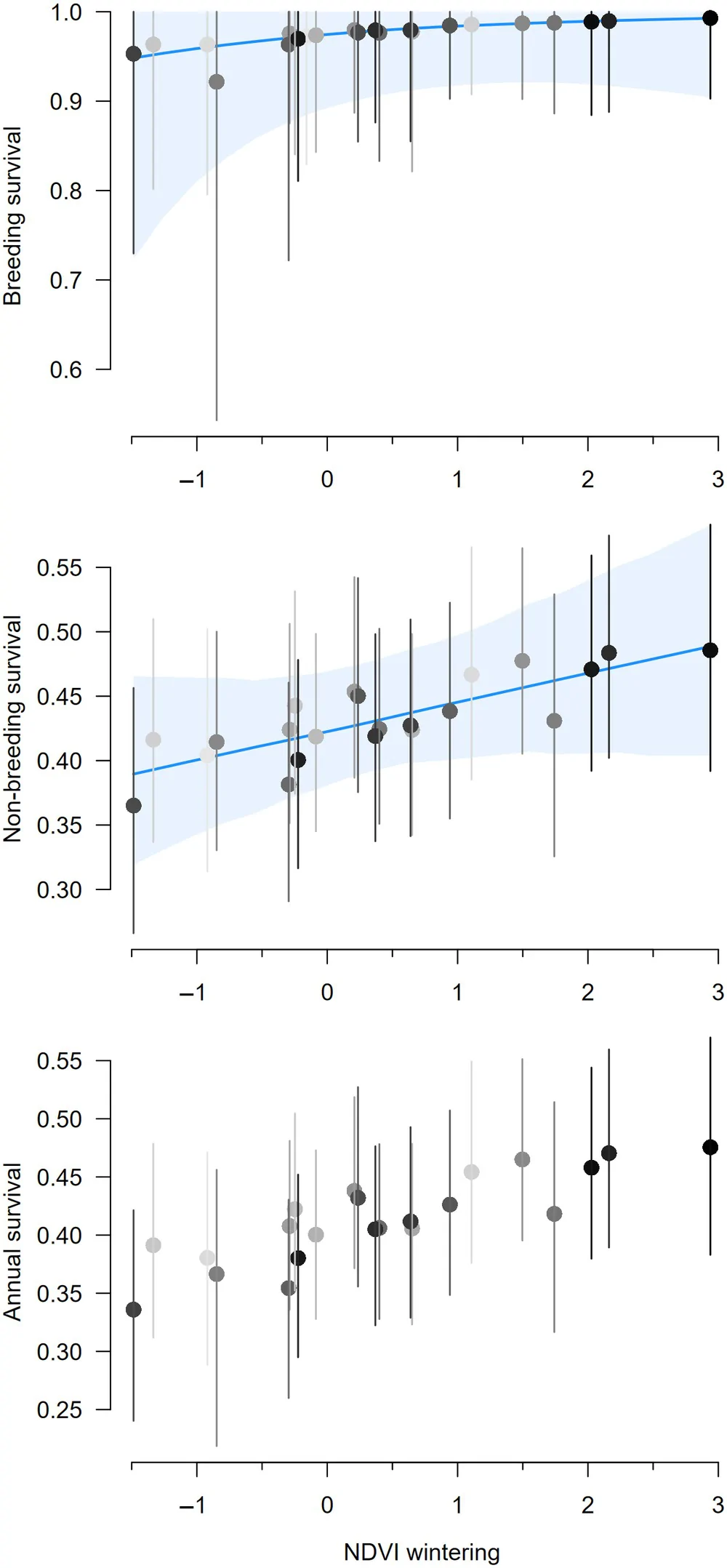
Breeding, non‐breeding and annual survival probabilities of adult hoopoes in relation to the scaled Normalized Difference Vegetation Index (NDVI) values from the wintering grounds estimated from the overall best model (*h* ^*B*^(NDVI^w^), *h* ^*N*^(NDVI^w^), Table 3). Dots show posterior medians and the vertical line the 95% highest density intervals. The blue lines show posterior medians of the predictions under the best model, with shaded areas depicting the 95% highest density intervals. The colors indicate the years with a gradient from light gray (2002) to black (2024).

In the second step, we compared all the different combinations of the selected environmental covariates (Table 3). The model that included the effect of winter NDVI on both non‐breeding and breeding period mortality hazard rates was the overall best model based on WAIC. The winter NDVI negatively affected both seasonal mortality hazards (Table 3); thus, survival increased with increasing greenness in the wintering grounds (Figure 7). While the variation in winter NDVI resulted in a strong effect size for non‐breeding and annual survival, its delayed (carry‐over) effect on breeding survival was marginal. The second‐best model included an additional effect of breeding weather on the mortality hazard rate during the breeding period. This effect was negative; therefore, dry and warm conditions during the breeding period, which are beneficial for reproductive success (Arlettaz, Schaub, Reichlin, et al., 2010), also tended to reduce mortality.

**TABLE 3:** Model selection results for the impact of environmental covariates (NDVI^w^: winter NDVI; PC: first principal component of breeding temperature and precipitation) on mortality hazards during the breeding (*h* ^*B*^) and the non‐breeding period (*h* ^*N*^).

Model	lppd	pWAIC	ΔWAIC	β_1_	*P*_1_	β_2_	*P*_2_	β_3_	*P*_3_
*h* ^*B*^(t), *h* ^*N*^(t)	−12826.56	70.05	1.42	…	…	…	…	…	…
*h* ^*B*^(t), *h* ^*N*^(NDVI^w^)	−12825.31	71.68	0.38	−0.067 (−0.160; 0.015)	0.93	…	…	…	…
*h* ^*B*^(NDVI^w^), *h* ^*N*^(t)	−12826.31	70.87	0.75	−0.511 (−1.637; 0.672)	0.90	…	…	…	…
*h* ^*B*^(t), *h* ^*N*^(PC)	−12825.95	71.51	1.31	−0.048 (−0.139; 0.047)	0.85	…	…	…	…
***h*** ^***B***^ **(NDVI** ^**w**^ **), *h*** ^***N***^ **(NDVI** ^**w**^ **)**	**−12825.30**	**71.50**	**0.00**	**−0.433** **(−1.622; 0.812)**	**0.78**	**−0.062** **(−0.153; 0.022)**	**0.93**	…	…
*h* ^*B*^(NDVI^w^), *h* ^*N*^(NDVI^w^ + PC)	−12825.01	72.12	0.65	−0.403 (−1.774; 0.984)	0.73	−0.062 (−0.148; 0.026)	0.93	−0.050 (−0.143; 0.046)	0.85
*h* ^*B*^(NDVI^w^), *h* ^*N*^(PC)	−12826.09	71.66	1.89	−0.499 (−1.831; 0.809)	0.79	−0.054 (−0.153; 0.039)	0.88	…	…
*h* ^*B*^(t), *h* ^*N*^(NDVI^w^ + PC)	−12825.78	71.73	1.40	−0.065 (−0.153; 0.017)	0.94	−0.047 (−0.139; 0.044)	0.85	…	…

*Note*: For each model we give: model name; log predictive density (lppd); estimated effective number of parameters (pWAIC); difference in the widely applicable Akaike information criterion between the current and the best model (ΔWAIC); estimated regression coefficients (posterior median and 95% highest density interval, β); and probabilities that the effect is negative (*P*). In case of multiple regression coefficients, they are listed in their order of appearance in the model notation. All models included annual random effects for the two hazard rates, and the model for the encounter rate was always the same as described in the text. The model with the lowest ΔWAIC value is presented in bold.

## DISCUSSION

We developed a continuous‐time capture–recapture model to estimate seasonal mortality using data collected in only one season. Based on our simulations, we conclude that seasonal survival can be estimated accurately, provided that the temporal pattern of encounter rates during the data collection period is correctly specified and that arrival and departure dates are approximately known. Given that much capture–recapture data on migratory species are collected exclusively during the breeding period, this model enables annual mortality to be decomposed into components during breeding and outside breeding, and their annual variation to be assessed. This is a significant step forward in developing full annual cycle models for migratory species based on existing data.

Migratory hoopoes experienced a much higher risk of mortality during the non‐breeding period, which included the two migratory journeys, than during the breeding period. This finding is consistent with the results of other studies on different migratory bird species, which have shown that stationary periods during the breeding and non‐breeding periods are less risky than nonstationary migratory periods (Cooper et al., 2024; Paxton et al., 2017; Robinson et al., 2020; Rushing et al., 2017; Sillett & Holmes, 2002). Non‐breeding survival was found to mirror annual survival very closely, suggesting that annual variation in non‐breeding survival is important for population dynamics. We also found that annual variation in seasonal survival depended on the average NDVI in the wintering grounds, a correlate of food supply sensu lato. This relationship was strong during the non‐breeding period and weaker, though still apparent, during the breeding period. Therefore, there was evidence of a carry‐over effect of environmental conditions during wintering that extended to survival during breeding, which has rarely been observed thus far (Cooper et al., 2024; Rockwell et al., 2017).

There is increasing evidence that seasonal migrations are the most dangerous phases in the life cycle of migratory birds (Cooper et al., 2024; Newton, 2025; Robinson et al., 2020), which is corroborated by the present findings. Although we could not directly estimate mortality during migration based on our data, there is manifest indirect evidence. In effect, in study models where survival probabilities of stationary breeding and wintering periods could be estimated, they typically appear similar (Cooper et al., 2024; Klaassen et al., 2014; Paxton et al., 2017; Rushing et al., 2017; Swift et al., 2020). Therefore, assuming that the hazard rate in hoopoes during the wintering period is identical to the hazard rate estimated for the breeding period, the mortality hazard rate during migration would be 102.39 × 10^−4^ (HDI: 91.36 × 10^−4^–144.51 × 10^−4^), resulting in survival probabilities of 0.62 (HDI: 0.57–0.70) and 0.72 (HDI: 0.69–0.79) for the autumn and spring migration, respectively. Mortality hazard would then be 65.2 (HDI: 0.9–1154.7) times greater during migration than during the stationary periods, with 93% of the annual mortality occurring during migration (Table 1). This value is higher than in other studies but would be reduced if the mortality hazard rate during wintering was larger than during breeding. As winter NDVI has a stronger effect on mortality hazards than the environmental conditions on the breeding grounds, it is likely that mortality is higher during the stationary non‐breeding period than during the breeding period.

Our results are consistent with the prediction that primary production (here measured via NDVI) on the wintering grounds affects seasonal survival; however, we cannot infer the exact cause of mortality. The Sahelian wintering grounds receive seasonal rainfall from July to September (Zwarts et al., 2009), enabling the development and growth of vegetation and plant‐dwelling invertebrates (Schlaich et al., 2016). Thus, when the hoopoes arrive at their wintering grounds in September after their autumn migration, they find an abundance of prey. Later, a long period of drought starts in the Sahel due to a lack of rainfall, annihilating ground vegetation cover and having a massive negative impact on invertebrate communities. Consequently, foraging opportunities continue to deteriorate as the season progresses. In years when precipitation remains particularly low even during the rainy season, the Sahel region dries out faster, with invertebrate prey supply presumably declining earlier and more quickly than under normal weather conditions. Reduced prey availability may result in a decline of body reserves prior to spring migration (Cooper et al., 2024; Ouwehand et al., 2023). This could lead to an increase in non‐breeding mortality and/or a later departure from the wintering grounds (Studds & Marra, 2011). Insufficient body reserves could thus limit the ability to cope with the physically demanding spring migration, ultimately affecting survival. As arrival on the breeding grounds in time to occupy high‐quality territories is important (van Wijk et al., 2017), birds that depart late from the wintering grounds may attempt to compensate by migrating faster, which could in turn further compromise their survival (Dossman et al., 2023). In conclusion, it is likely that increased mortality occurring under conditions of lower primary production operates via reduced food intake and subsequently depleted body reserves. However, it should be noted that this study was unable to determine whether mortality occurs on the wintering grounds or during spring migration.

Breeding survival itself was also negatively affected by the level of primary production on the wintering grounds, which points to carry‐over effects. Carry‐over effects on breeding survival have been rarely observed in other bird species (but see Cooper et al., 2024). Nevertheless, this indirect effect was markedly weaker than the direct impact of primary productivity on non‐breeding survival. Once again, one can only speculate about the underlying mechanism. Being short‐lived, hoopoes should invest in their current rather than their future reproduction (Stearns, 1992). Several lines of evidence suggest that hoopoes prioritize current reproduction over their own survival. There are strong dependencies between reproduction, survival, and seasonal environmental conditions. Firstly, cold and wet conditions during the breeding period negatively affect both reproductive success and adult survival (see Table 3), suggesting that hoopoes are ready to sacrifice their own lives for their offspring (Plard et al., 2018). Secondly, following unfavorable conditions on the wintering grounds, individuals that survive the spring migration may arrive with depleted energy reserves. While this appears to have no effect on current reproduction (Souchay et al., 2018), it clearly impacts apparent survival (this study), that is, either true survival and/or the emigration from the study population.

The modeling approach developed in this study is appealing because it can be applied to other extant longitudinal capture–mark–recapture datasets. The main idea is to accurately estimate annual survival and survival during breeding for making inferences on non‐breeding survival. In principle, this requires the capture of individuals shortly after arrival on and again shortly before departure from the breeding grounds (Robinson et al., 2020), but this is often not feasible in practice. Instead, one can rely on the captures or encounters that occur throughout the breeding season, as demonstrated in this study. We conclude that the continuous‐time capture–recapture model presented here can be efficiently used to estimate seasonal survival in various demographic settings. This paves the way for complete annual cycle models of population dynamics (Marra et al., 2015). Using existing longitudinal data acquired over multiple years allows us to investigate temporal variation in seasonal survival and include it in an integrated population model where the dynamics of a breeding population are modeled with seasonal survival probabilities (Rushing et al., 2017; Schaub & Kéry, 2022).

However, our simulations show that particular attention must be paid to the specification of encounter rates, as this involves a somewhat arbitrary categorization. Comparing models using different categorization is a possibility to find an appropriate solution (see Appendix S1: Section S3). Additionally, knowledge about approximate arrival and departure dates is necessary, which can be obtained through direct observations or by using encounter dates of marked individuals if no geolocator data are available as in our study. The simulation results suggest it is preferable to define the breeding season length as too short rather than too long, since a shorter period does not introduce bias into the breeding mortality hazard rate. The model only permits the distinction between breeding and non‐breeding survival; it does not allow disentangling migratory and wintering survival. Overcoming this obstacle would require encounter data to be collected on the winter grounds. Ultimately, continuous‐time models applied to data collected during both the breeding and non‐breeding periods could then be combined in a similar way to discrete‐time capture–recapture models (Rushing, 2019; Rushing et al., 2017). The novel modeling approach presented here offers a valuable tool for understanding species' demography during a complete annual cycle, which is crucial for comprehending population dynamics and guiding conservation action.

## AUTHOR CONTRIBUTIONS

Michael Schaub and Ellen C. Martin conceived the ideas, Michael Schaub analyzed the data and led the writing, Irmi Zwahlen collected the data, and all authors contributed critically to the drafts and gave final approval for publication.

## CONFLICT OF INTEREST STATEMENT

The authors declare no conflicts of interest.

## Supporting information


Appendix S1.

## Data Availability

Data and code (Schaub et al., 2026) are available in Zenodo via the vogelwarte.ch Open Repository and Archive at https://doi.org/10.5281/zenodo.20021072.
