# Supplementary material for: Estimating seasonal survival of migratory birds using continuous‐time capture–recapture models
Source: Ecol Appl. 2026 Sep 8;36(6):e70301. doi: 10.1002/eap.70301 (PMC13551070; doi:10.1002/eap.70301)
Supplement: Supplementary file 1 [file EAP-36-e70301-s001.pdf]

Appendix S1

Estimating seasonal survival of migratory birds using continuous-time capture–recapture models

Michael Schaub, Raphaël Arlettaz, Irmi Zwahlen, Ellen C. Martin

Ecological Applications

Section S1: Extended results from simulation studies

Section S2: Derivation of the environmental variables

Section S3: Patterns of encounter rates within the breeding season

Section S4: Priors used in the hoopoe case study

Section S5: Estimates from the basic model (h^B(t), h^N(t))

Section S1: Extended results from simulation studies

S1. General parameter accuracy and identifiability

We simulated data where the mortality hazard rates during breeding and non-breeding were variable over the year, as were the encounter rates during breeding. This data was analysed with a model that perfectly matched the data simulation, i.e., all underlying assumptions were met in this set-up. See the main text for more details about the simulation parameters and Figure 3 (main text) for the first results.

In order to assess the performance of the parameter estimates, we computed the relative bias of the mortality hazard rates during breeding and non-breeding and of the encounter rates. Box plots of relative bias show that it is virtually zero for all parameters (Figure S1).

We also converted the simulated data into binary annual capture–recapture data. From encounter histories, we checked whether an individual was detected at least once during the breeding period and used this information to construct capture histories on an annual basis. We analysed these data using a Cormack-Jolly-Seber (Lebreton et al., 1992) model to estimate annual survival and recapture probabilities. We then calculated the annual survival probabilities from the seasonal mortality hazard rates and compared them with the probabilities obtained using the classical CJS model. This was motivated by the desire to quantify how well the developed model performs in terms of estimating annual survival compared to the standard CJS model. From Figure S2 we can see that the annual estimates from the continuous time model are unbiased, whereas there was a fairly substantial negative bias in survival from the CJS model. The bias may be due to the 'pooling' of the data, i.e. their transformation from the continuous to the discrete scale. In addition, the estimation uncertainties (SD of the estimates) from the continuous time model are much lower than that from the discrete time CJS model. This difference is probably due to the fact that less information is used in the discrete-time CJS model.

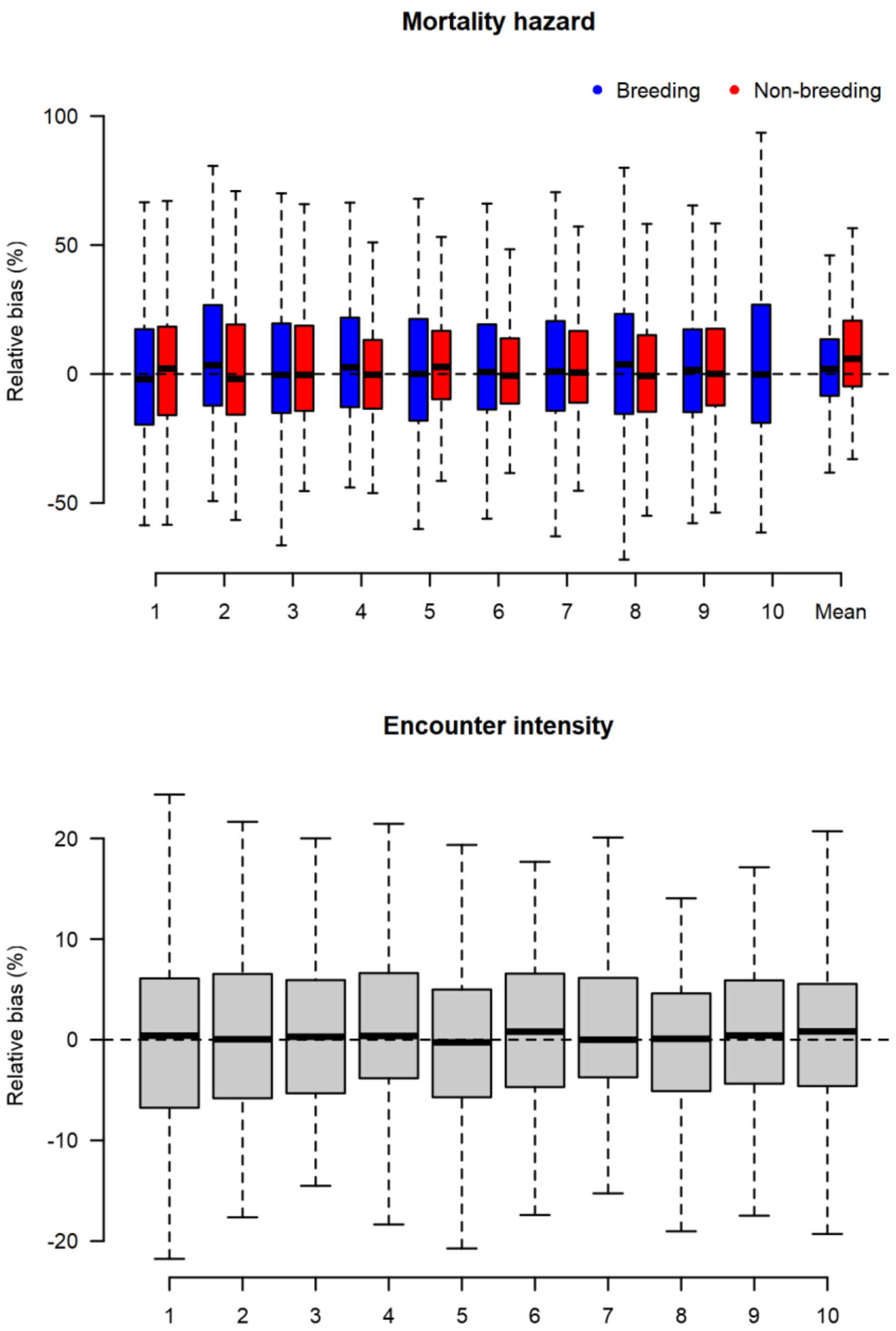

Figure S1. Box plots of the relative bias (of the posterior means) of year-specific and mean seasonal mortality hazards (top panel) and encounter rates (bottom panel) under a model with random time effects based on 200 simulated datasets. The numbers on the x-axis refer to years.

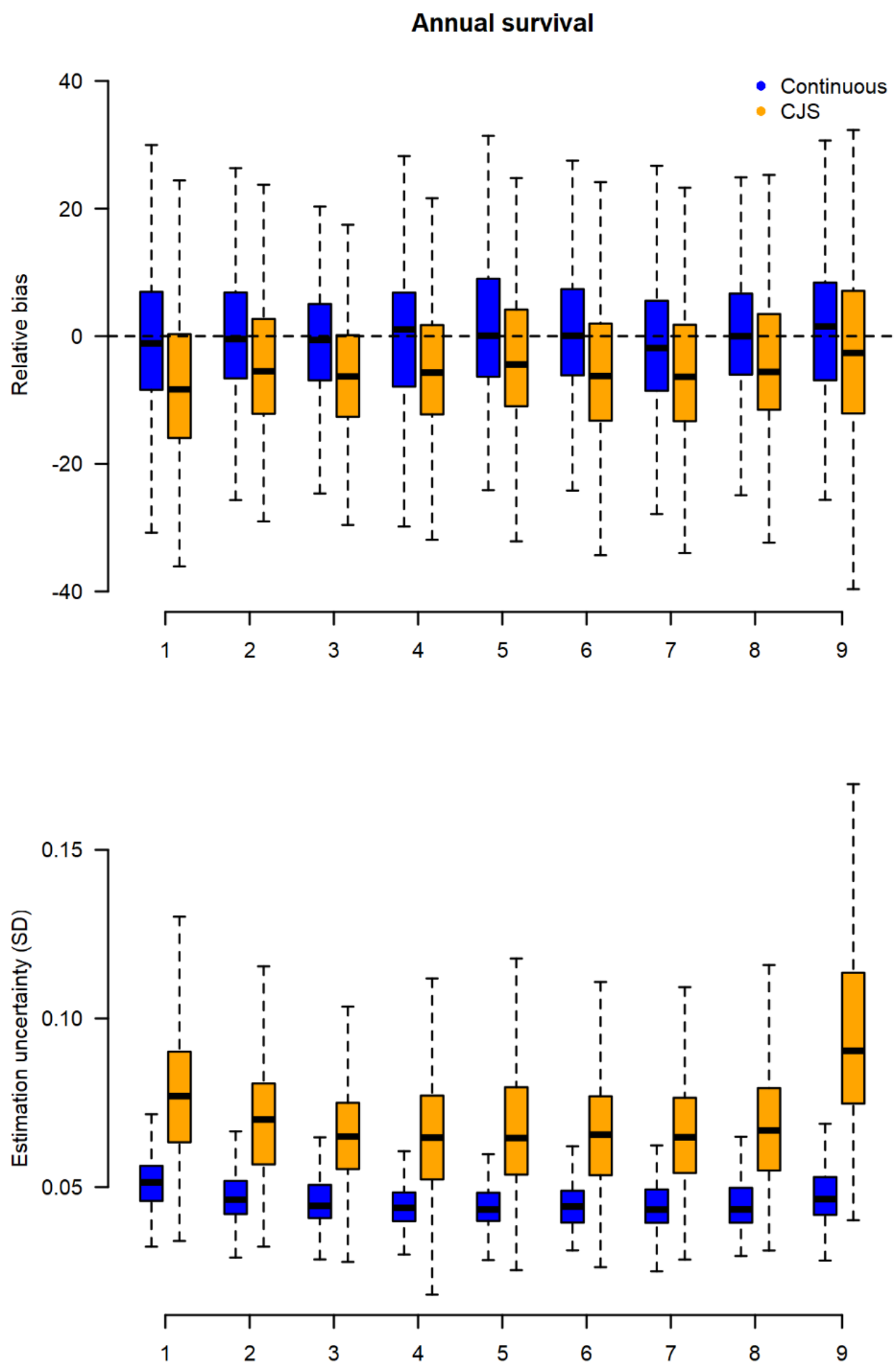

Figure S2. Boxplots of relative bias (upper panel) and of estimation uncertainty (SD, lower panel) of year-specific annual survival under the continuous and the discrete time CJS models.

Conclusion

The continuous-time model with seasonal mortality hazard rates produces identifiable and accurate parameter estimates even if no data are collected during one season. This conclusion holds if the model assumptions are met. We also see that the annual estimates from the continuous-time model are unbiased, whereas the discrete-time CJS model produces biased estimates of annual survival.

S2. Model performance under violating assumptions

S2.1. Mis-specified temporal pattern in encounter rates during the breeding season

The second simulation study assessed the performance of the model to estimate parameters when the temporal pattern of encounter rates were incorrectly specified in the model, i.e. if the data generation model did not match the data analysis model. We used two scenarios. First, we assumed that the encounter rate was higher (p_1 = 0.015) in the first half of the breeding period than in the second half (p_2 = 0.005). Second, we assumed the reverse pattern, that encounter was lower in the first period (p_1 = 0.005) than in the second (p_2 = 0.015). In all scenarios we further assumed that the mortality hazard was lower during breeding (h_1 = 0.002) than during non-breeding (h_2 = 0.0025), but that there was no annual variation. For each simulation, we assumed the marking of 100 individuals annually over a period of 10 years. The breeding period was set at 132 days and the non-breeding period at 233 days. We created 200 simulation replicates.

We then used four models to analyze the resulting data sets. In all models, the hazard rates were estimated with two different parameters that were constant over time. For the encounter rates, four models were used that differed in the temporal pattern of encounter rates during the breeding period. First, we assumed a single value (constant encounter throughout the breeding period). Second, we assumed that the breeding period was divided in half and each half had its own encounter rate. This model corresponded to the data generation model. Thirdly, the breeding period was divided into three time intervals of equal length and different encounter rates were estimated for each of them. Finally, the breeding period was divided into four time intervals of equal length and different encounter rates were estimated for each. Models 1 and 3 violated the model assumptions, model 1 more than model 3. Models 2 and 4 did not violate the model assumptions, but model 4 was less parsimonious than model 2.

Figure S3 shows the posterior means of all mortality hazards and encounter rates under both scenarios. It appears that the estimates of mortality hazards are biased if the temporal pattern in encounter rates is not correctly captured (models 1 and 3). The largest bias occurs in model 1, which assumes that encounter rates are constant over the entire breeding period, when in fact they differed between the first and the second half of the breeding period. The direction of the bias depends on the scenario. If in reality the encounter rate is higher in the first than in the second interval of the breeding period (scenario 1), the breeding mortality hazard is positively biased and the non-breeding mortality hazard is negatively biased. The reverse pattern is found when encounter rate is actually lower in the first than in the second interval (scenario 2). When three time intervals of encounter rates are specified in the model, the pattern of bias in the mortality hazards is the same, but the bias is much reduced. The mortality hazard estimates are unbiased in both scenarios if either the correct number of encounter rates are specified (model 2) or a larger number of encounter rates are used that capture the correct temporal pattern (model 4).

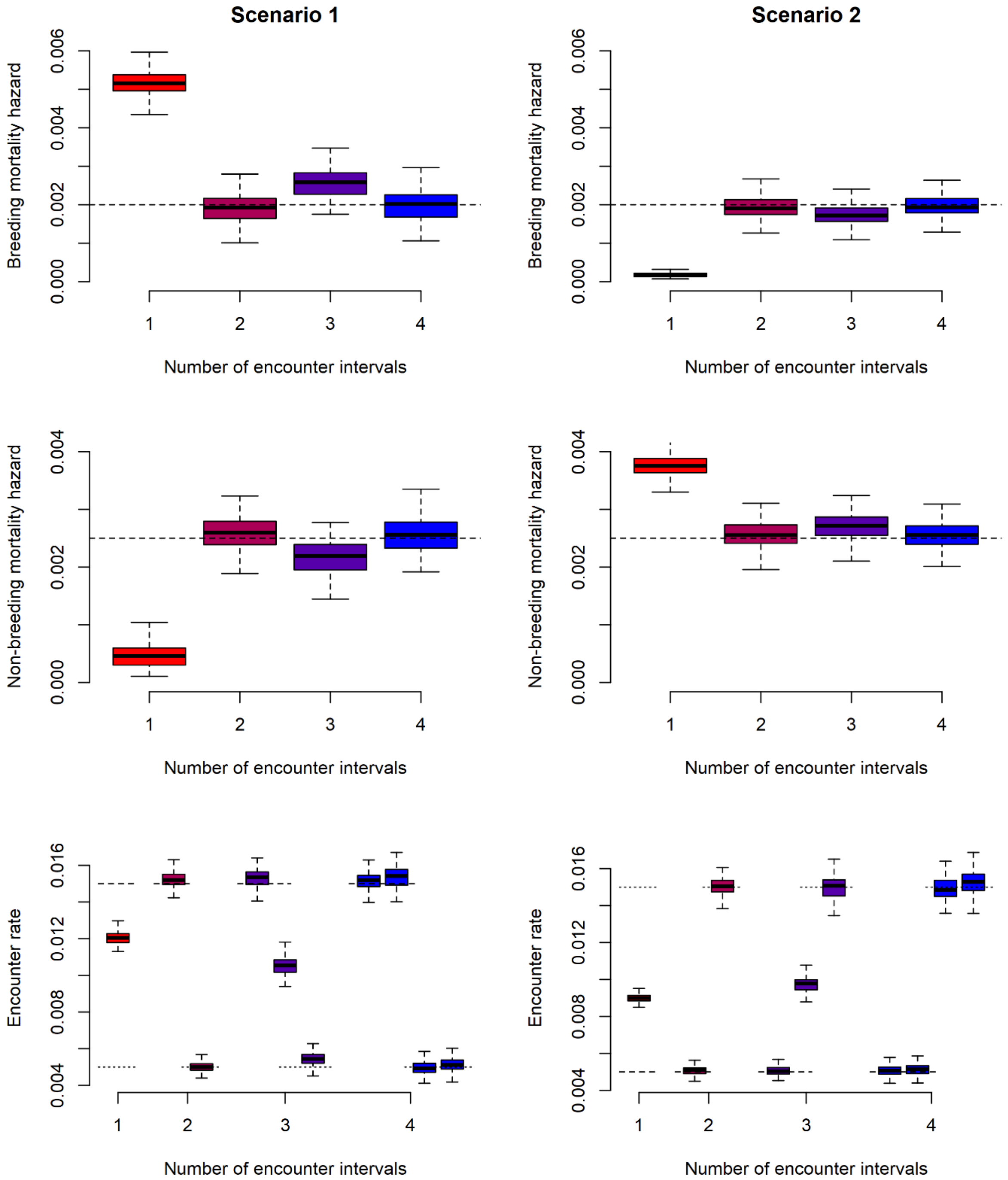

Figure S3. Box plots of posterior means (across simulations) of seasonal mortality hazards and encounter rates obtained from models considering different numbers of encounter intervals during breeding (4 models represented by 4 colors). The panels in the left column are obtained under scenario 1, where the true encounter rate is higher in the first than in the second interval. The panels in the right column are obtained under scenario 2, where the true encounter rate is lower in the first than in the second interval.

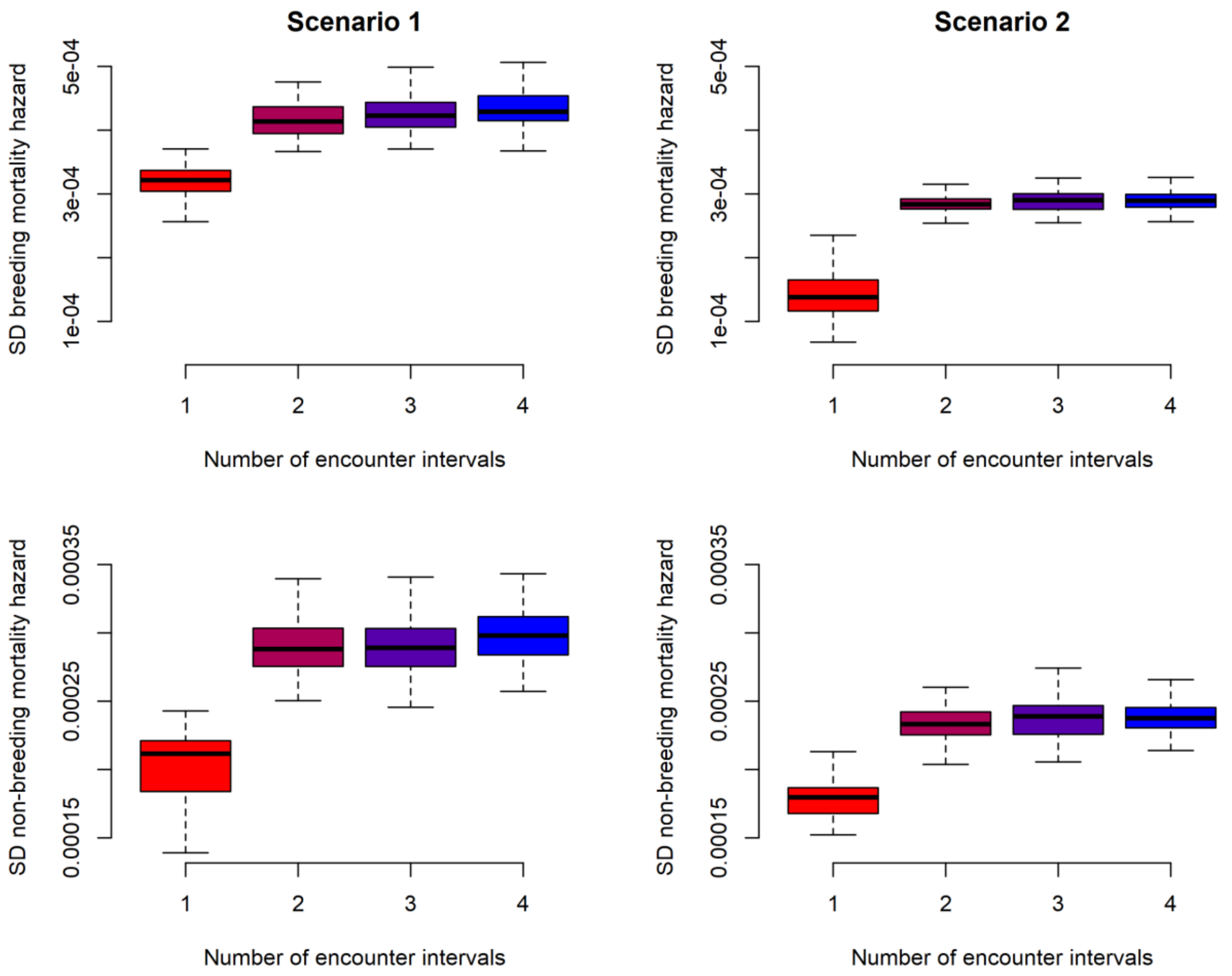

Figure S4. Box plots of posterior standard deviations (across simulations) of seasonal mortality hazards obtained from models considering different numbers of encounter intervals during breeding (4 models represented by 4 colors). The panels in the left column are obtained under scenario 1, where the true encounter rate is higher in the first than in the second interval. The panels in the right column are obtained under scenario 2, where the true encounter rate is lower in the first than in the second interval.

Figure S4 shows box plots of the posterior standard deviations (SD) of the mortality hazards. In general, as expected, the SD increases with the number of intervals during the breeding period considered. However, the main difference is when only one or more intervals are considered, but there is not much change when more intervals are considered. Interestingly, mortality hazard rates are more accurately estimated when the encounter rate is higher in the second interval of the breeding period. This suggests that there is an advantage

when encounter is high just before the start of the non-breeding period when no encounters occur.

Conclusion

Capturing the correct temporal pattern of encounter rates during the breeding period is important for accurate estimation of seasonal mortality risks. The bias will be reduced if a model is used that allows temporal flexibility in encounter rates (more intervals), even if the temporal pattern is not entirely correct. Therefore, it is recommended to use enough temporal resolution in encounter rates. The price in terms of reduced precision (Figure S4) seems to be small (at least in our simulation setup). However, computation time increases significantly with the number of intervals considered.

S2.2. Mis-specified duration of the breeding period

The model assumes uniform and perfectly known arrival and departure dates for all individuals, resulting in fixed durations of breeding and non-breeding periods. However, actual arrival and departure dates vary among individuals and may be uncertain. The third simulation therefore examines the performance of the model to estimate parameters when: 1) arrival and departure dates vary (two levels), 2) arrival is assumed later and departure earlier than reality with the result that the specified breeding period is too short compared to reality, and 3) arrival is assumed earlier and departure later with the result that the specified breeding period is too long compared to reality. We also included a basic scenario when all model assumptions are met, i.e., when arrival and departure dates do not vary among individuals and are perfectly known.

We used a similar set-up to that described in Section S1: S2.1 for these simulations: The mortality hazard rate during breeding was h_1 = 0.002, while it was h_2 = 0.0025 during the non-breeding period. The encounter rate during breeding was p = 0.01. For a total of 10 years, 100 individuals were newly marked each year, resulting in a data set containing 1,000 individuals. For scenarios where arrival and departure dates varied among individuals, we generated these dates by drawing from a normal distribution. The mean values were set as the average arrival and departure dates, corresponding to a mean breeding period of 132 days and a non-breeding period of 233 days. The standard deviation was either 5 days (for moderate variability) or 10 days (for strong variability). In the moderate scenario, 95% of birds arrived and departed within a 20-day window; in the strong variation scenario, this window extended to 40 days. For the scenarios with assumed too short or too long breeding periods, the survival was simulated for breeding and non-breeding period lengths of 132 and 233 days, respectively. When a too short breeding period was assumed, the encounter dates were collected only in the interval 15 days

after arrival until 15 days before departure, hence in a period that was 30 days shorter than the true breeding period. For the scenario when a too long breeding period was assumed, encounters were simulated for the complete true breeding period of 132 days.

The models used for analysing the simulated data sets assumed constant mortality hazard rates between years during both breeding and non-breeding periods, as well as for encounter rates. The boundaries marking the end and beginning of the breeding and non-breeding periods were determined by the mean arrival and departure dates for both the basic and variable scenarios. In the scenario with a too short breeding period, the dates were set to 15 days after the mean arrival and 15 days before the mean departure, resulting in a specified breeding period of 102 days and a non-breeding period of 263 days. For the scenario with an extended breeding period, the dates were set at 15 days before the mean arrival and 15 days after the mean departure, thereby lengthening the specified breeding period to 162 days and reducing the non-breeding period to 203 days. A total of 200 simulations were run for each of the five scenarios.

The main results are reported in the paper (Fig. 5). When arrival and departure dates vary among individuals around a mean that remains constant over time, there is no bias in the estimated parameters, but slightly increased uncertainty. When the breeding period is assumed to be too short than it is in reality, the estimate of mortality hazard rate during breeding is unbiased, but with increased uncertainty. The encounter rate also remains unbiased, but its estimation uncertainty increases. The increased estimation uncertainty in both parameters is a direct consequence of the reduced duration over which the two continuous processes are measured. When the breeding period is assumed to be too long than it is in reality, the breeding mortality hazard rate becomes positively biased, and the non-breeding mortality hazard rate negatively biased. In addition, the encounter rate is negatively biased.

We calculated annual survival probabilities from the seasonal mortality hazard rates taking into account the duration of the assumed seasonal periods. These annual survival probabilities demonstrate minimal bias among all scenarios (Fig. S5).

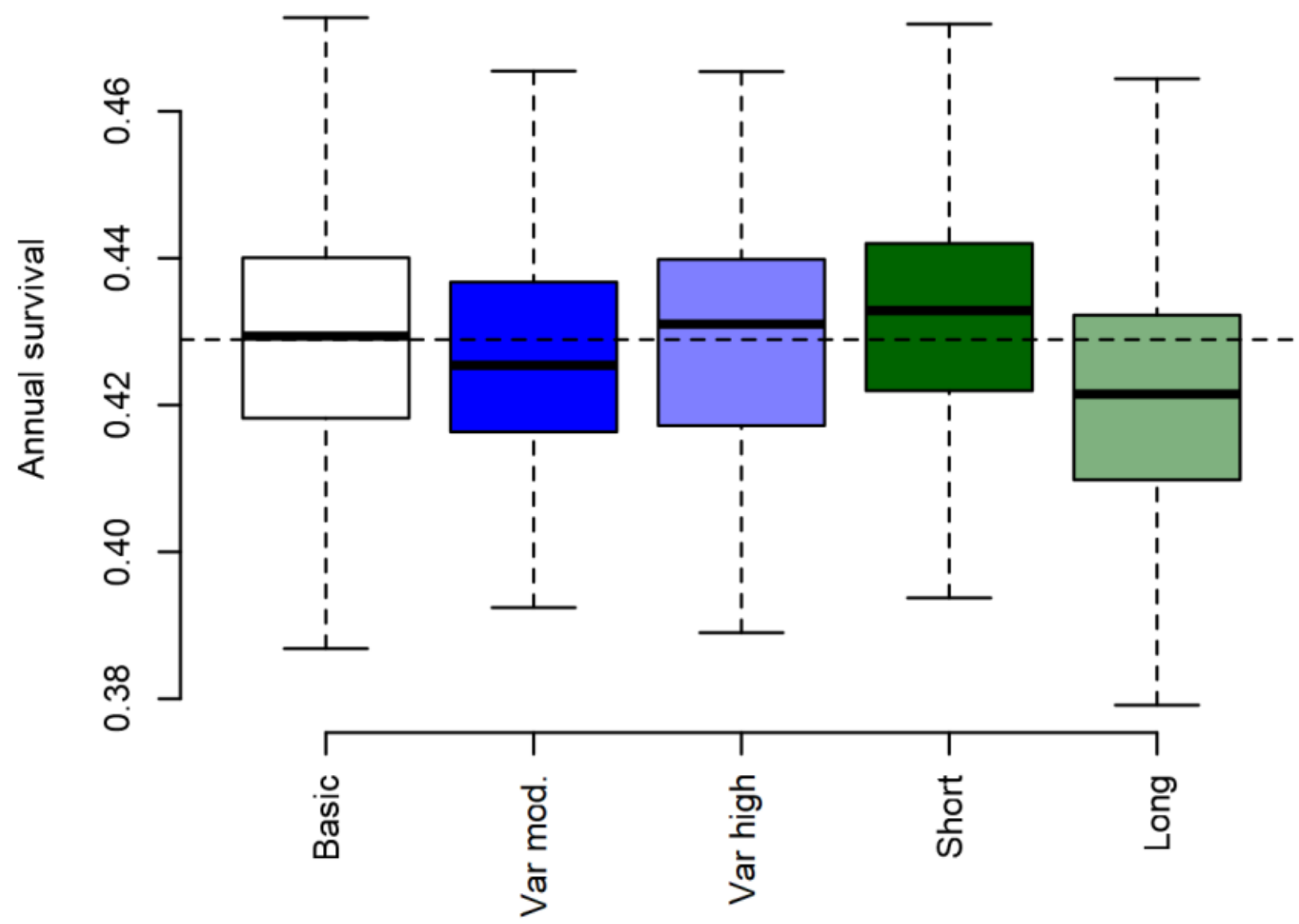

Figure S5. Box plots show the posterior means of annual survival probabilities across five scenarios: Basic (all assumptions met), Var. mod (moderate variability in arrival/departure dates), Var. strong (strong variability in arrival/departure dates), Short (breeding period assumed too short), and Long (breeding period assumed too long). The dashed line represents the true annual survival probability.

Conclusion

Violations of the assumptions that arrival and departure dates are identical and precisely known for all individuals can impact parameter estimates. However, variability among individuals proved to be a minor concern. The most pronounced bias occurred when the breeding period was assumed to be longer than in reality, whereas assuming a shorter breeding period primarily increased parameter uncertainty. For practical applications, it is therefore more prudent to select a breeding period that may be slightly too short rather than one that is excessively long.

Section S2: Derivation of the environmental variables

Breeding ground

Daily temperature and precipitation data from 1981 to 2024 were obtained from the weather station Sion (46°13'N, 7°20'E; Federal Office of Meteorology and Climatology MeteoSwiss. Precipitation observations from Sion (SIO) station, including rka150d0 and rretntd0 parameters: Daily and hourly totals). The breeding period was defined beginning on April 5 and extending until August 15. We calculated the average daily temperature and daily precipitation across all days in the breeding period, which yielded one mean value per year for each covariate. As we aimed for a single variable that characterized weather conditions on the breeding grounds, we performed a principal component analysis (PVA) using the calculated annual temperature and precipitation and used the first component as the environmental variable for the breeding ground. The first principal component (PC) explained 62.2% of the variance. It was positively correlated with temperature (r=0.79) and negatively with the amount of precipitation (r=-0.79), hence a high PC value indicates a warm and dry breeding year.

Wintering ground

To characterize environmental conditions on the wintering grounds (Fig. 2), we used MODIS-derived NDVI data spanning 1981–2024. Raster layers (two NDVI composites per month) were clipped using polygons defined from known locations of use of individual hoopoes from geolocator data (Schaub et al., 2025). NDVI values were extracted from September through March, excluding the early September and late March composites to align with the wintering period (September 14–March 12). For each wintering period, defined as September through December in year t and January through March in year $t+1$, we computed the mean NDVI values across all raster layers within the polygons, which yielded a seasonal NDVI mean value per year. This variable was then standardized and denoted as $NDVI^w$.

Migratory stopover grounds

We quantified NDVI on stopover grounds during spring (March 6–April 1) and autumn (August 22–September 10) migration whose locations are known from geolocator data (Fig. 2). As done for the wintering grounds, raster files were clipped, and layers corresponding to the migration periods were included. For each migration period, we calculated the mean NDVI per year. Spring migration NDVI values were attributed to the year of arrival on breeding grounds (e.g., March 1990 values assigned to 1990), while autumn migration values were assigned to the year of departure. The two NDVI variables were then standardized and denoted as $NDVI^s$ (spring migration stopover) and $NDVI^a$ (autumn migration stopover).

Section S3: Pattern of encounter rates within the breeding season

Continuous-time models assume that the mortality hazard rate and the encounter rate are constant within chosen intervals. Across these intervals the values may change and therefore we can model temporal variation. For our hoopoe study it is clear a priori that we assume constant mortality hazard rates during the breeding and the non-breeding periods, but they may vary between years. There are no encounters of hoopoes during the non-breeding period and hence the encounter rate is constant and fixed to zero. Yet, during the breeding period it is likely that the encounter rate is not constant, because not all hoopoes are multiple brooding and hence the encounter rate is expected to be lower at the end of the breeding period than earlier. Yet the temporal pattern within a breeding period is unknown, but the model requires that we must assign one with some temporal resolution. Here, we investigate the consequences of assigning different temporal resolutions of encounter rates for our hoopoe data set.

Firstly, we compared models with different temporal resolutions of the encounter rate during the breeding period. For simplicity, we assumed that encounter rates were constant across years. As in the models described in the paper, we used different temporal random effects for the mortality hazard rates during the breeding and non-breeding periods (model h^B(t), h^N(t)). The following six temporal resolutions had even interval lengths and were used for encounter rates during the 132-day breeding period:

- 1 time interval (132 days)
- 2 time intervals (each 66 days)
- 3 time intervals (each 44 days)
- 4 time intervals (each 33 days)
- 6 time intervals (each 22 days)
- 12 time intervals (each 11 days)

Based on the estimated encounter rates from the most flexible model with 12 time intervals, we added two temporal resolutions with unequal interval lengths:

- 3 time intervals (33 days, 66 days, 33 days)
- 4 time intervals (22 days, 22 days, 55 days, 33 days)

We compared these eight temporal resolutions for the encounter rates. We examined the estimates of the target parameters, the mortality hazard rates. From our simulation study we know that once the temporal pattern in the encounter rates is flexible enough, the patterns in the mortality hazard rates are not expected to change with increasing number of time intervals. Therefore, we can choose the temporal resolution at which there is no further change in the mortality hazards (the most parsimonious one).

In a second step, we adopted the identified temporal resolution and added annual variation in different ways: an additive model (p{time interval + year}) and an interactive model (p{time

interval x year}). We treated the year as a fixed effect and compared the three models using WAIC.

Results

Figure S6 shows the year-specific mortality hazard rates during the breeding (upper panel) and non-breeding (lower panel) periods, using data with different temporal resolutions for encounter rate during the breeding period. These hazard rates show the same temporal patterns, and they differ little once more than four time intervals during the breeding period are considered. This can be seen more clearly when the mean hazard rates and survival probabilities are plotted (Figure S7). When there are only three time intervals, the hazard rates differ considerably; however, when at least four time intervals are chosen, the resulting estimates of mortality hazards are very similar. In particular when the four time intervals of unequal length are used (22, 22, 55 and 33 days), the hazard rates are almost identical to those obtained when more than four time intervals are used.

Figure S8 shows the estimated encounter rates. The most flexible model, which has 12 time intervals, best shows the seasonal pattern of encounter rates. In general, the encounter rate appears to be low at the beginning of the breeding period, increasing strongly before remaining fairly high throughout much of the breeding period, and then declining again. This pattern is very well captured when four time intervals with unequal lengths are considered, but not when a lower number of time intervals are used. Following the principle of parsimony, the preferred model is the four-time interval resolution with unequal durations.

The preference is not only driven by the increased accuracy of the parameter estimates, but also by the fact that computation time increases significantly with the number of time intervals considered, as shown below:

Number of time intervals	Computation time
1	7.9h
2	10.3h
3	14.4h
3 unequal [33, 66, 33]	12.5h
4	18.6h
4 unequal [22, 22, 55, 33]	13.7h
6	25.9h
12	51.6h

MCMC specifications: 15,000 iterations, 5,000 burn-in, 3 chains

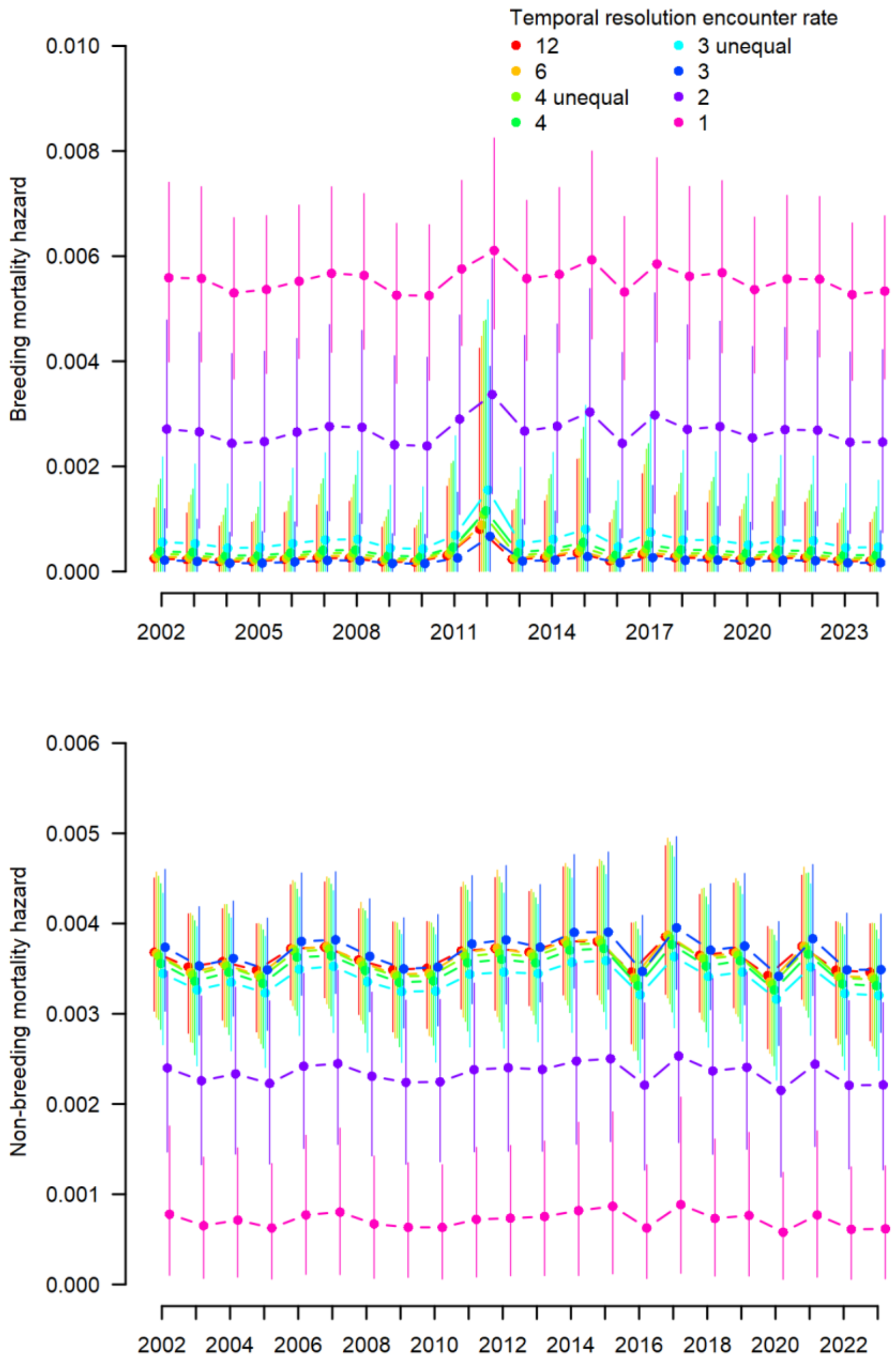

Figure S6. Comparison of estimated mortality hazards during the breeding (upper panel) and non-breeding (lower panel) periods between different temporal resolutions for encounter rates during the breeding period.

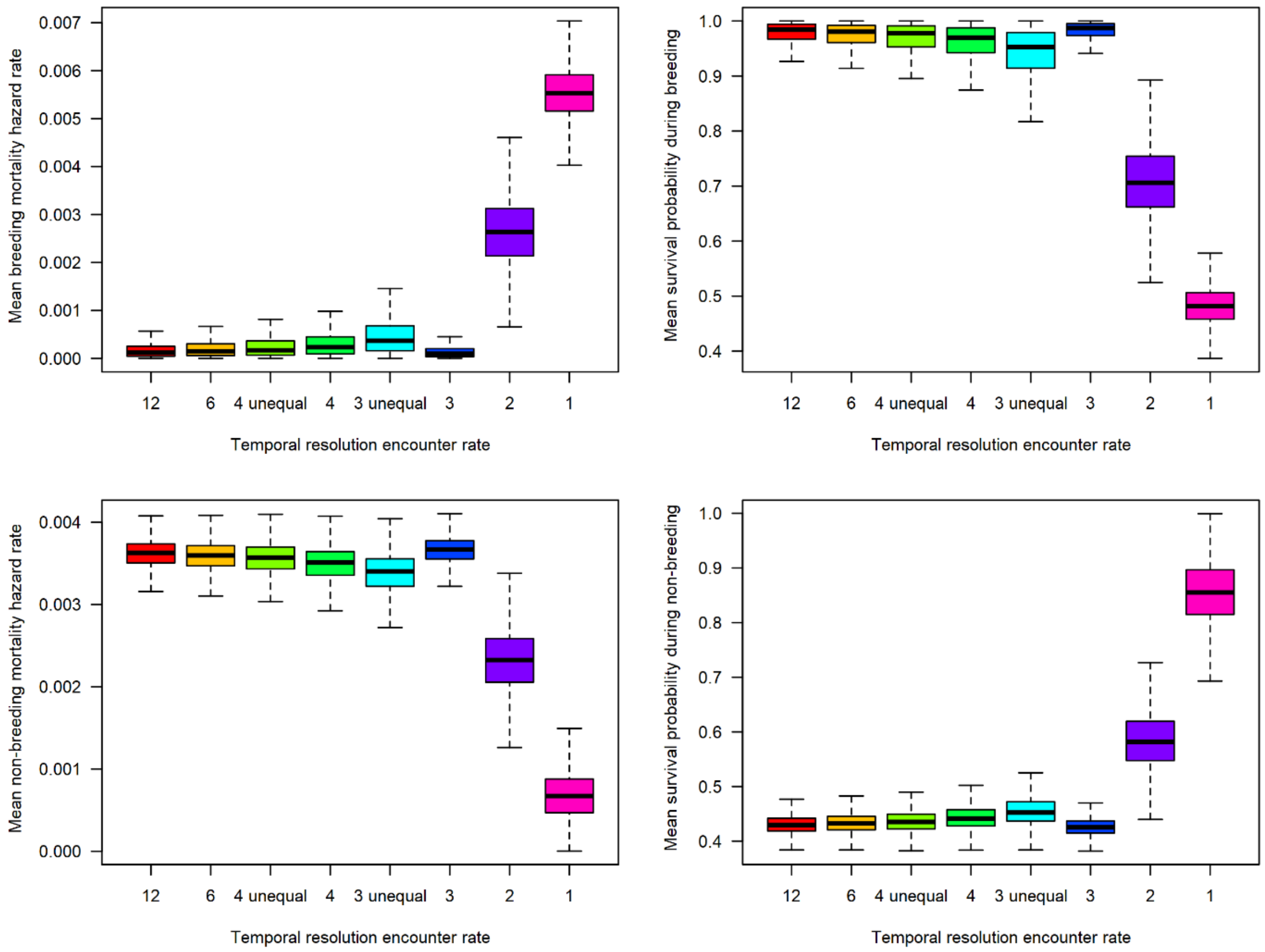

Figure S7. Comparison of estimated mean mortality hazards (upper left panel) and seasonal survival (upper right panel) during the breeding period and mean mortality hazards (lower left panel) and seasonal survival (lower right panel) during the non-breeding period between different temporal resolutions for encounter rates during the breeding period.

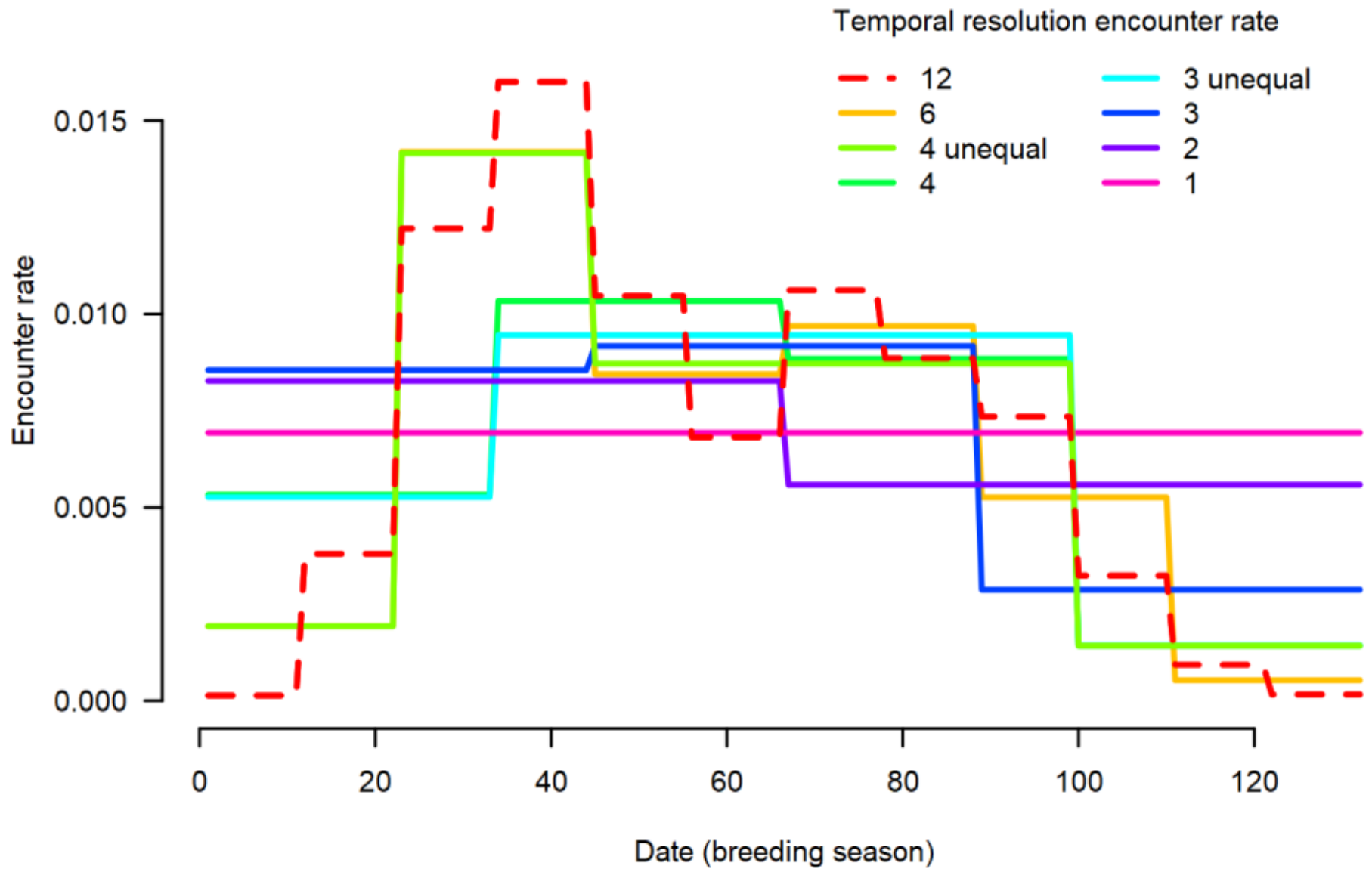

Figure S8. Estimated encounter rates under the different models, and how they apply to the seasonality.

Given that we aim for four time intervals with unequal lengths (22, 22, 55 and 33 days) we test different models that also include annual variation in encounter rate and perform model selection. The model with an interaction between time intervals and year is by far the best (Table S1). The resulting mortality hazards are shown in Figure S9. While the estimates from the first three models are nearly the same, the encounter model with the interaction of time intervals and year produced quite different hazard rates. Figure S10 also shows the resulting encounter rates of the four time intervals over the 23 study years. While the encounter rates during the second and third time interval did not show an obvious trend, the rate during the first time interval clearly increased from 2018 when we started to use alphanumeric color rings and motion-triggered cameras to identify birds.

Table S1. Model selection results for the impact of annual variation in encounter rates. The following is provided for each model: model name; log predictive density (lppd); difference in the Watanabe-Akaike information criterion between the current and the best model (ΔWAIC); estimated effective number of parameters (pWAIC); computation time (25,000 iterations, 5,000 burn-in, 3 chains).

Model	lppd	ΔWAIC	pWAIC	Computation time
p(time interval)	-12937.99	104.6	11.84	13.7h
p(time interval+ year)	-12919.52	104.2	30.08	47.1h
p(time interval x year)	-12826.56	0.00	70.95	68.1h

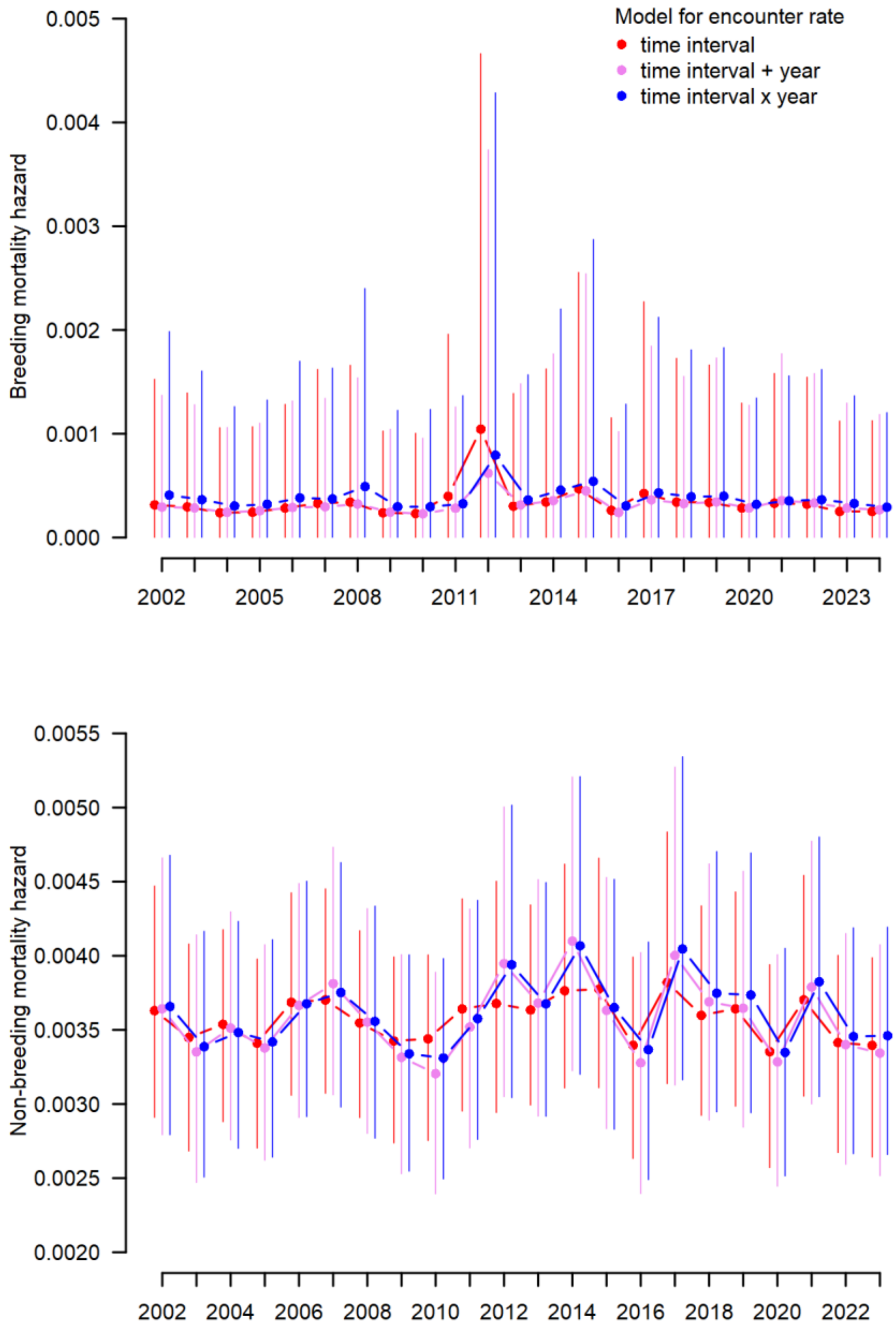

Figure S9. Comparison of estimated mortality hazards during the breeding and non-breeding periods between different models of encounter rates during the breeding season. The temporal resolution is always 4 time intervals (22, 22, 55 and 33 days).

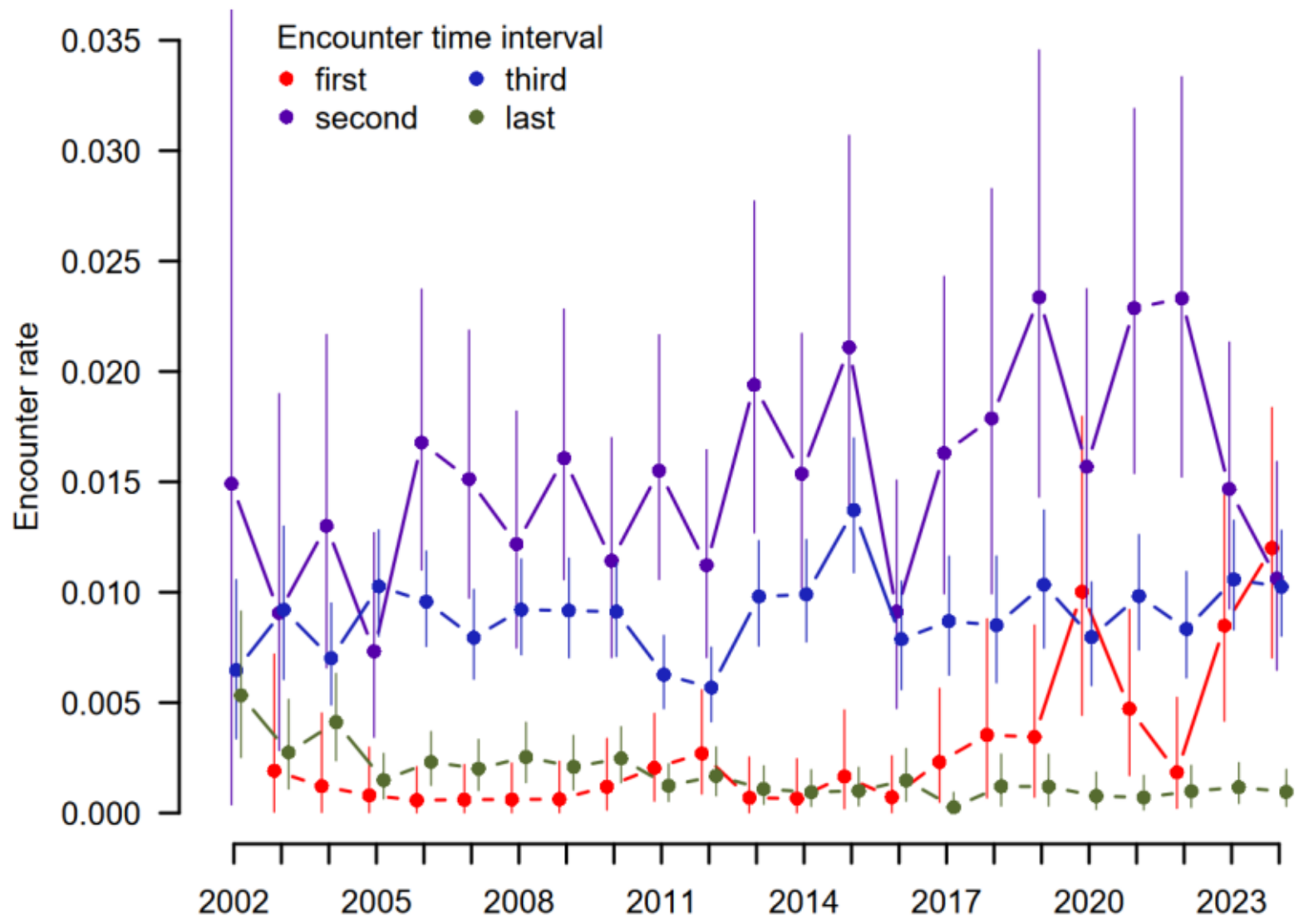

Figure S10. Encounter rates in the four different encounter time intervals in the breeding period over the years based on the best model (time interval x year).

Section S4

Table S2. Priors used in the hoopoe case study.

Parameter	Prior	Distribution name
Mean mortality hazard rate	$\bar{h} \sim U(0,0.1)$	Uniform
Temporal standard deviation of mortality hazards	$\sigma \sim hC(0,0.05)$	half Cauchy
Slope for environmental variables	$\beta \sim Normal(0,1)$	Normal
Encounter rates	$p \sim U(0,0.1)$	Uniform

Section S5: Estimates from the basic model (h^B(t), h^N(t))

Table S3. Estimated parameters under model (h^B(t), h^N(t)) from the hoopoe study. The following are given for each parameter: posterior means and medians; the limits of the 95% credible interval (l95 and u95); the *R*-hat value (a diagnostic of MCMC chain convergence); the significance of the parameter; and the period and year to which it belongs.

Parameter	mean	median	l95	u95	Rhat	Significance	Period	Year
ht[1]	0.0004081	0.0002173	0	0.0014249	0.997	hazard rate	Breeding	2002
ht[2]	0.0003649	0.0002126	0	0.0012262	1.000	hazard rate	Breeding	2003
ht[3]	0.0003044	0.0001842	0	0.0010004	0.998	hazard rate	Breeding	2004
ht[4]	0.0003203	0.0001959	0	0.0010576	0.998	hazard rate	Breeding	2005
ht[5]	0.0003815	0.0002184	0	0.001323	0.996	hazard rate	Breeding	2006
ht[6]	0.0003720	0.0002137	0	0.0012744	1.000	hazard rate	Breeding	2007
ht[7]	0.0004903	0.0002582	0	0.0017943	1.001	hazard rate	Breeding	2008
ht[8]	0.0002965	0.0001812	0	0.0009865	0.997	hazard rate	Breeding	2009
ht[9]	0.0002964	0.0001799	0	0.0009717	1.001	hazard rate	Breeding	2010
ht[10]	0.0003257	0.000194	0	0.0010894	0.999	hazard rate	Breeding	2011
ht[11]	0.0007934	0.0003323	0	0.0033719	1.003	hazard rate	Breeding	2012
ht[12]	0.0003636	0.0002101	0	0.0012542	1.001	hazard rate	Breeding	2013
ht[13]	0.0004565	0.0002402	0	0.0016551	0.998	hazard rate	Breeding	2014
ht[14]	0.0005417	0.0002618	0	0.0021237	1.000	hazard rate	Breeding	2015
ht[15]	0.0003053	0.0001847	0	0.0010171	0.998	hazard rate	Breeding	2016
ht[16]	0.0004309	0.0002303	0	0.001562	0.996	hazard rate	Breeding	2017
ht[17]	0.0003927	0.0002198	0	0.0013602	1.001	hazard rate	Breeding	2018
ht[18]	0.0003986	0.0002221	0	0.0013765	0.996	hazard rate	Breeding	2019
ht[19]	0.0003196	0.0001910	0	0.0010551	0.998	hazard rate	Breeding	2020
ht[20]	0.0003540	0.0002048	0	0.0012047	0.999	hazard rate	Breeding	2021
ht[21]	0.0003644	0.0002125	0	0.0012413	1.002	hazard rate	Breeding	2022
ht[22]	0.0003280	0.0001965	0	0.0010856	0.999	hazard rate	Breeding	2023
ht[23]	0.0002923	0.0001810	0	0.0009675	1.002	hazard rate	Breeding	2024
ht[24]	0.0036581	0.0036384	0.0027330	0.0046101	1.002	hazard rate	Non-breeding	2002-2003
ht[25]	0.0033877	0.0034148	0.0025076	0.0041651	1.000	hazard rate	Non-breeding	2003-2004
ht[26]	0.0034824	0.0034976	0.0027021	0.0042321	1.000	hazard rate	Non-breeding	2004-2005
ht[27]	0.0034190	0.0034410	0.0026841	0.0041421	1.003	hazard rate	Non-breeding	2005-2006
ht[28]	0.0036763	0.0036665	0.0029093	0.0044916	0.996	hazard rate	Non-breeding	2006-2007
ht[29]	0.0037520	0.0037294	0.0029854	0.0046337	1.000	hazard rate	Non-breeding	2007-2008
ht[30]	0.0035573	0.0035642	0.0027625	0.0043234	1.001	hazard rate	Non-breeding	2008-2009
ht[31]	0.0033391	0.0033638	0.0025673	0.0040205	1.003	hazard rate	Non-breeding	2009-2010
ht[32]	0.0033102	0.0033412	0.0024926	0.0039779	1.001	hazard rate	Non-breeding	2010-2011
ht[33]	0.0035762	0.0035816	0.0027816	0.0043930	1.000	hazard rate	Non-breeding	2011-2012
ht[34]	0.0039397	0.0038967	0.0030147	0.0049815	0.997	hazard rate	Non-breeding	2012-2013
ht[35]	0.0036761	0.0036684	0.0029099	0.0044812	0.998	hazard rate	Non-breeding	2013-2014

ht[36]	0.0040674	0.0040139	0.0031648	0.0051457	1.000	hazard rate	Non-breeding	2014-2015
ht[37]	0.0036499	0.0036399	0.0028207	0.0045028	0.999	hazard rate	Non-breeding	2015-2016
ht[38]	0.0033673	0.0033983	0.0025288	0.0041245	1.000	hazard rate	Non-breeding	2016-2017
ht[39]	0.0040449	0.0039614	0.0030833	0.0052201	1.000	hazard rate	Non-breeding	2017-2018
ht[40]	0.0037475	0.0037173	0.0028966	0.0046395	1.000	hazard rate	Non-breeding	2018-2019
ht[41]	0.0037356	0.0037099	0.0028931	0.0046329	1.001	hazard rate	Non-breeding	2019-2020
ht[42]	0.0033480	0.0033764	0.0025602	0.0040872	1.000	hazard rate	Non-breeding	2020-2021
ht[43]	0.0038245	0.0037815	0.0030138	0.0047526	0.999	hazard rate	Non-breeding	2021-2022
ht[44]	0.0034556	0.0034736	0.0026706	0.0041908	0.999	hazard rate	Non-breeding	2022-2023
ht[45]	0.0034609	0.0034795	0.0026754	0.0042042	0.999	hazard rate	Non-breeding	2023-2024
mean.h[1]	0.0002977	0.0002098	0	0.0008882	0.997	mean hazard rate	Breeding	
mean.h[2]	0.0035928	0.0036123	0.0031272	0.0040051	1.003	mean hazard rate	Non-breeding	
sigma.h[1]	0.9335242	0.7482803	0.0237400	2.409345	0.990	temp. variation hazard	Breeding	
sigma.h[2]	0.1166603	0.1132065	0.0086404	0.221579	1.002	temp. variation hazard	Non-breeding	
pt[1]	0.0501138	0.0498985	0.0010187	0.0959973	1.001	encounter rate	Breeding	2002
pt[2]	0.0149179	0.0103778	9.00E-07	0.0444025	1.000	encounter rate	Breeding	2002
pt[3]	0.0064690	0.0063040	0.0030869	0.0101278	1.001	encounter rate	Breeding	2002
pt[4]	0.0053292	0.0051580	0.0022826	0.0088056	1.000	encounter rate	Breeding	2002
pt[5]	0.0019150	0.0012954	0	0.0058157	1.001	encounter rate	Breeding	2003
pt[6]	0.0090645	0.0083929	0.0020609	0.0173376	1.000	encounter rate	Breeding	2003
pt[7]	0.0092036	0.0090963	0.0058462	0.0127068	1.003	encounter rate	Breeding	2003
pt[8]	0.0027465	0.0026135	0.0008559	0.0047562	1.000	encounter rate	Breeding	2003
pt[9]	0.0012162	0.0008409	0	0.0037129	1.002	encounter rate	Breeding	2004
pt[10]	0.0130097	0.0126071	0.0059288	0.0206441	1.000	encounter rate	Breeding	2004
pt[11]	0.0070117	0.0069482	0.0047916	0.0093709	1.000	encounter rate	Breeding	2004
pt[12]	0.0041130	0.0040308	0.0022261	0.0061098	1.001	encounter rate	Breeding	2004
pt[13]	0.0008010	0.0005542	0	0.0023814	1.002	encounter rate	Breeding	2005
pt[14]	0.0073249	0.0070539	0.0029357	0.0119449	0.998	encounter rate	Breeding	2005
pt[15]	0.0102674	0.0102181	0.0078889	0.0126658	0.999	encounter rate	Breeding	2005
pt[16]	0.0014918	0.0014250	0.0005757	0.0025664	1.002	encounter rate	Breeding	2005
pt[17]	0.0005795	0.0004018	1.00E-07	0.0017214	1.001	encounter rate	Breeding	2006
pt[18]	0.0167795	0.0165691	0.0106519	0.0232347	1.000	encounter rate	Breeding	2006
pt[19]	0.0095722	0.0095345	0.0073966	0.0116964	1.000	encounter rate	Breeding	2006
pt[20]	0.0023120	0.0022609	0.0011982	0.0035776	0.998	encounter rate	Breeding	2006
pt[21]	0.0006043	0.0004234	1.00E-07	0.0017887	0.997	encounter rate	Breeding	2007
pt[22]	0.0151257	0.0148757	0.0093351	0.0212134	1.001	encounter rate	Breeding	2007
pt[23]	0.0079481	0.0078939	0.0059430	0.0099798	0.998	encounter rate	Breeding	2007
pt[24]	0.0020084	0.0019516	0.0009737	0.0032344	1.001	encounter rate	Breeding	2007
pt[25]	0.0006162	0.0004262	2.00E-07	0.0018404	1.000	encounter rate	Breeding	2008
pt[26]	0.0121859	0.0119670	0.0070664	0.0176048	0.999	encounter rate	Breeding	2008
pt[27]	0.0092112	0.0091649	0.0070462	0.0113649	1.000	encounter rate	Breeding	2008

pt[28]	0.0025395	0.0024738	0.0013039	0.0039558	1.001	encounter rate	Breeding	2008
pt[29]	0.0006262	0.0004335	0	0.0018662	1.002	encounter rate	Breeding	2009
pt[30]	0.0160716	0.0158775	0.0102622	0.0223763	0.999	encounter rate	Breeding	2009
pt[31]	0.0091680	0.0091137	0.0069545	0.0114519	1.000	encounter rate	Breeding	2009
pt[32]	0.0020885	0.0020213	0.0009463	0.0033740	0.999	encounter rate	Breeding	2009
pt[33]	0.0011952	0.0009930	2.31E-05	0.0028698	1.000	encounter rate	Breeding	2010
pt[34]	0.0114292	0.0112452	0.0067446	0.0164876	1.002	encounter rate	Breeding	2010
pt[35]	0.0091176	0.0090859	0.0070691	0.0112898	1.000	encounter rate	Breeding	2010
pt[36]	0.0024730	0.0024161	0.0013152	0.0038139	1.000	encounter rate	Breeding	2010
pt[37]	0.0020393	0.0018752	0.0003218	0.0040175	1.001	encounter rate	Breeding	2011
pt[38]	0.0155062	0.0153061	0.0103026	0.0211873	1.000	encounter rate	Breeding	2011
pt[39]	0.0062680	0.0062322	0.0046557	0.0079405	1.000	encounter rate	Breeding	2011
pt[40]	0.0012313	0.0011764	0.0004763	0.0021241	0.997	encounter rate	Breeding	2011
pt[41]	0.0026958	0.0025013	0.0006537	0.0051377	1.001	encounter rate	Breeding	2012
pt[42]	0.0112236	0.0110146	0.0066639	0.0160120	1.001	encounter rate	Breeding	2012
pt[43]	0.0056830	0.0056341	0.0040613	0.0073930	1.002	encounter rate	Breeding	2012
pt[44]	0.0016825	0.0016182	0.0007304	0.0028697	1.002	encounter rate	Breeding	2012
pt[45]	0.0006881	0.0004819	0	0.0020512	0.998	encounter rate	Breeding	2013
pt[46]	0.0193943	0.0191411	0.0121719	0.0270249	1.000	encounter rate	Breeding	2013
pt[47]	0.0098037	0.0097493	0.0074932	0.0122158	1.002	encounter rate	Breeding	2013
pt[48]	0.0010899	0.0010303	0.0002958	0.0019649	1.001	encounter rate	Breeding	2013
pt[49]	0.0006560	0.0004546	0	0.0019909	0.998	encounter rate	Breeding	2014
pt[50]	0.0153714	0.0152343	0.0100209	0.0214582	0.999	encounter rate	Breeding	2014
pt[51]	0.0099033	0.0098488	0.007644	0.0122519	1.000	encounter rate	Breeding	2014
pt[52]	0.0009463	0.0008825	0.0002277	0.0017856	0.998	encounter rate	Breeding	2014
pt[53]	0.0016593	0.0013728	4.56E-05	0.0039967	1.002	encounter rate	Breeding	2015
pt[54]	0.0210968	0.0207009	0.0130271	0.0297754	1.000	encounter rate	Breeding	2015
pt[55]	0.0137170	0.0136336	0.0107237	0.016786	1.000	encounter rate	Breeding	2015
pt[56]	0.0010058	0.0009419	0.0002601	0.0019073	0.997	encounter rate	Breeding	2015
pt[57]	0.0007166	0.0004940	1.00E-07	0.0021188	0.998	encounter rate	Breeding	2016
pt[58]	0.0091179	0.0088339	0.0043254	0.0144308	0.997	encounter rate	Breeding	2016
pt[59]	0.0078722	0.0078072	0.0055315	0.0104045	1.000	encounter rate	Breeding	2016
pt[60]	0.0014827	0.0013998	0.0004335	0.0027115	1.003	encounter rate	Breeding	2016
pt[61]	0.0023115	0.0020505	0.0002096	0.0049686	1.002	encounter rate	Breeding	2017
pt[62]	0.0163031	0.0160334	0.0094557	0.0236168	0.998	encounter rate	Breeding	2017
pt[63]	0.0086881	0.0086008	0.0060685	0.0113762	1.001	encounter rate	Breeding	2017
pt[64]	0.0002601	0.0001821	0	0.0007650	0.999	encounter rate	Breeding	2017
pt[65]	0.0035377	0.0031177	0.0003583	0.0077271	0.997	encounter rate	Breeding	2018
pt[66]	0.0178710	0.0174461	0.009312	0.0272549	1.002	encounter rate	Breeding	2018
pt[67]	0.0085132	0.0084263	0.0057829	0.0114807	1.001	encounter rate	Breeding	2018
pt[68]	0.0012072	0.0011071	0.0002072	0.0024123	0.999	encounter rate	Breeding	2018
pt[69]	0.0034460	0.0030487	0.0003207	0.0073767	0.998	encounter rate	Breeding	2019
pt[70]	0.0233646	0.0229201	0.0137059	0.0336217	1.000	encounter rate	Breeding	2019
pt[71]	0.0103425	0.0102533	0.0073137	0.0134984	1.000	encounter rate	Breeding	2019

pt[72]	0.0011965	0.0010946	0.0001869	0.0023691	1.001	encounter rate	Breeding	2019
pt[73]	0.0100228	0.0095803	0.0038389	0.0169264	0.999	encounter rate	Breeding	2020
pt[74]	0.0156872	0.0153776	0.0087629	0.0229915	0.999	encounter rate	Breeding	2020
pt[75]	0.0079732	0.0079091	0.0056931	0.0103609	1.001	encounter rate	Breeding	2020
pt[76]	0.0007699	0.0006843	9.07E-05	0.0016618	1.001	encounter rate	Breeding	2020
pt[77]	0.0047272	0.0044671	0.0013295	0.0085584	0.999	encounter rate	Breeding	2021
pt[78]	0.0228685	0.022586	0.0151724	0.0315510	1.000	encounter rate	Breeding	2021
pt[79]	0.0098256	0.0097533	0.0073298	0.0125385	1.002	encounter rate	Breeding	2021
pt[80]	0.0007071	0.0006299	6.14E-05	0.0014930	1.000	encounter rate	Breeding	2021
pt[81]	0.0018404	0.0015278	4.08E-05	0.0044861	1.000	encounter rate	Breeding	2022
pt[82]	0.0233165	0.0230049	0.0148121	0.0327036	1.000	encounter rate	Breeding	2022
pt[83]	0.0083354	0.0082775	0.0060670	0.0108520	1.000	encounter rate	Breeding	2022
pt[84]	0.0009840	0.0009045	0.0001743	0.0019611	0.999	encounter rate	Breeding	2022
pt[85]	0.0084860	0.0081851	0.0036172	0.013568	1.001	encounter rate	Breeding	2023
pt[86]	0.0146772	0.0144677	0.0091059	0.0210740	0.998	encounter rate	Breeding	2023
pt[87]	0.0105825	0.0105217	0.0081483	0.0130732	1.000	encounter rate	Breeding	2023
pt[88]	0.0011701	0.0011055	0.0003253	0.0021012	1.002	encounter rate	Breeding	2023
pt[89]	0.0120008	0.0117418	0.0068784	0.0180974	0.999	encounter rate	Breeding	2024
pt[90]	0.0106219	0.0104304	0.006143	0.0154569	1.000	encounter rate	Breeding	2024
pt[91]	0.0102433	0.0101901	0.0079683	0.0127533	1.001	encounter rate	Breeding	2024
pt[92]	0.0009572	0.0008903	0.0002165	0.0017984	1.000	encounter rate	Breeding	2024
pt[93]	0	0	0	0	NA	encounter rate	Non-breeding	

References

Lebreton, J.-D., Burnham, K. P., Clobert, J., Anderson, D. R., 1992. Modeling survival and testing biological hypothesis using marked animals: a unified approach with case studies. Ecol. Monogr. 62, 67–118

Schaub, M.; Arlettaz, R.; Bauer, S.; van Wijk, R.E.; Liechti, F.; Bächler, E.; Capilla-Lasheras, P.; Nussbaumer, R. (2025): GeoLocator Data Package: Swiss Hoopoes. https://zenodo.org/records/15260024
